# Supplementary material for: Innovative Techniques for Infection Control and Surveillance in Hospital Settings and Long-Term Care Facilities: A Scoping Review
Source: Antibiotics (Basel). 2024 Jan 13;13(1):77. doi: 10.3390/antibiotics13010077 (PMC10812752; doi:10.3390/antibiotics13010077)
Supplement: Supplementary file 1 [file antibiotics-13-00077-s001.zip › antibiotics-2776611-supplementary.pdf]

Innovative techniques for infection control and surveillance in hospital setting and Long-Term Care Facilities: a Scoping review

Study protocol

1. Introduction

Healthcare-associated infections (HAIs) are among the major adverse events encountered by patients in any healthcare system, and are associated with significant mortality, morbidities and increasing healthcare cost.
Surveillance of HAIs and their risk factors is the cornerstone of programs for their control, but in its active form requires resources not always available; for example, a point prevalence survey- the most used measure of disease frequency to report HAI - can take up to 756-man hours or an estimated 1.5 fulltime equivalent (FTE) per 10,000 admissions.
Artificial Intelligence (AI) is being increasingly used in medicine for diagnostic purposes and may be applied to surveillance of HAIs. Automated surveillance can support (semi-automated) or completely replace (fully automated) manual surveillance thanks to the use of algorithms based on AI. AI can cope to the increased availability of data from different sources, which can be digitally stored in a single structured data system called data warehouse (DW).

2. Scope and objectives

The aim of this study is to explore the innovative tools for healthcare associated Infections surveillance and their applications in hospital settings and long-term care facilities. The second aim is highlighting the strengths and weaknesses of the innovative techniques compared to traditional one.

Scoping review: *Innovative tools for healthcare associated Infections surveillance among hospital and LTCF patients.*
Specific objectives:

i. Describe innovative tools for healthcare associated infections surveillance and their applications.
ii. Compare the innovative techniques to the traditional ones.

3. Proposed methodology

The scoping reviews will follow PRISMA-P guidelines. PRISMA-P ensures the transparency and completeness of a scoping review protocol.

3.1. Research questions

The research question for the scoping review has been formulated as:
What are the innovative surveillance techniques to identify HAI in hospital/LTCF?

Table S1. Scoping review PICOs framework question

PICOs Framework	
P - population	Patients admitted in hospital and LTCF

I - intervention	Innovative tools for infection control in hospital setting (e.g., ICU)
C - comparator	No comparator
O - outcome	Qualitative (primary): description of innovations in infection control and surveillance.<br>Quantitative (secondary): evaluation and comparison between Innovative Vs. Traditional in terms of accuracy.
S - setting	Hospital settings including all hospital wards and long-term facilities
S - study	Prospective, retrospective observational studies; Clinical studies including RCTs; Case series

3.2. Definitions

The following definitions are used for the purpose of this study:

– Innovative surveillance techniques: any new technology applied to the detection, control and surveillance of the HAI. Laboratory techniques and innovation related to clinical case management are excluded.
– Surveillance: the ongoing, systematic collection, analysis, and interpretation of health-related data with the purpose of preventing or controlling disease or injury, or of identifying unusual events of public health importance, followed by the dissemination and use of information for public health action
– LCTF: facilities which provide supervision and assistance in activities of daily living with medical and nursing services when required.
– HAI: Any infection which a patient contracts in a health-care institution.
– Hospitalised patient: is defined as an adult or paediatric patient admitted to any ward of a general, tertiary or university hospital.

3.3. Search strategy

The search strategies will be developed to include the following facets:

– Healthcare associated infections
– Hospital and LTCF settings
– Surveillance
– Innovation technologies

Proposed search limits:

- Language: All languages
- Time: 2018-2023
- Geographic: All worldwide countries

The literature search will be conducted using MEDLINE (Ovid), SCOPUS and Web of Science.

3.4. Records selection procedure

The results of searches will be downloaded and loaded in a bibliographic management software (Rayyan) and deduplication will be performed. The selection procedure will take place in three phases as described below. The selection of records will be performed by a team of reviewers and may be performed either via the independent and double screening

of all records or through an iterative double screening of a subset of records to achieve a concordance >95%, followed by single screening of the remaining records. Disagreements will be adjudicated by the group of reviewers. Studies excluded after assessment of the full text will be reported alongside reasons for exclusion.

1. Screening of title and abstract (first selection phase): this step will yield the articles that will be assessed in full text. In this first phase, titles of publications are screened based on the inclusion and exclusion criteria (see section 3.5). If the title is inconclusive, the abstract is read. If the title is inconclusive and no abstract is available, the full text of the article will be checked in the second selection step. Articles with titles and abstracts that suggest that they do not contain information relevant to the research objective will not be selected for full text assessment. Whenever it is clear that the article does not fulfil the eligibility criteria it will be excluded. In case of doubt, the article will be checked full text in the second selection step. Articles that have been excluded during screening of title and abstract will be stored in Microsoft Excel sheet.
2. Screening of full article (second selection phase): the articles selected during the first phase will be assessed in full text. PDF-files of the original articles will be downloaded and stored. Articles will be included if the reported information is relevant (based on the inclusion and exclusion criteria, see section 3.5). The reasons for exclusion of full text papers will be documented per article and summarised in an exclusion table. In this way the selection procedure is transparent and will assure reproducibility.
3. Screening during data-extraction phase: further scrutiny of the article during the data-extraction phase might lead to exclusion. For example, when articles make use of the same dataset and present identical outcome measures, the most recent or the most complete article will be included.

3.5. Inclusion and exclusion criteria

The draft list of inclusion and exclusion criteria is presented in Table 2. Should other relevant criteria emerge during the preliminary stage of the screening phase, the list will be adapted.

Table S2. Inclusion and exclusion criteria for scoping review

Variable	**Inclusion**	**Exclusion**
Study design/ type	• Randomised controlled trials (RCTs)<br>• Non-randomised, prospective comparative studies<br>• Prospective observational studies (e.g. cohort studies)<br>• Retrospective observational	• Narrative review<br>• Case reports<br>• Non-pertinent publication types (e.g. expert opinions, letters to the editor, editorials, comments, viewpoints)<br>• Animal studies<br>• Genetic studies, biochemistry or molecular studies

	studies (e.g. case-control studies)<br>• Cross-sectional studies<br>• Meta-analysis or systematic review<br>• Conference Proceedings	• Mathematical modelling studies<br>• *Study protocols*
Country	• All worldwide countries	• No exclusion
Study subject	• Innovative technologies for infection control and surveillance	• Other types of surveillance
Study population	• Hospitalised individuals (any hospital ward) and LTCF individuals	• Non-hospitalised individuals
Specific outcomes of interest	• Qualitative (primary): description of innovations in infection control<br>• Quantitative (secondary): evaluation and comparison of accuracy (innovative Vs. traditional methods)	• Outcomes not related to research question.

If high quality systematic reviews and/or meta-analysis are identified, their primary studies included will be included in the analysis.

3.6. Critical appraisal

Based on three methodological streams: quantitative (randomized group comparison, non-randomized group comparison and descriptive surveys), qualitative and mixed-methods (use of quantitative and qualitative methods), we will apply different quality appraisal tools as described in Table 3 below. For retrospective and prospective studies that are not valuable with standardised quality appraisal tool, we will use an adapted scale reported in the following supplementary materials.

The quality appraisal will be performed by one single reviewer.

Table S3. Quality Appraisal Tools

Type of Study Design	Quality Appraisal Tool	Link to the tool
Qualitative Study Design (including content Analysis)	Critical Appraisal Skills Programme (CASP)	http://www.casp-uk.net/#!casp-tools-checklists/c18f8
Mixed Methods	Mixed Methods Appraisal Tool (MMAT) – Version 2011	Pluye, P., Robert, E., Cargo, M., Bartlett, G., O'Cathain, A., Griffiths, F., Boardman, F., Gagnon, M.P., & Rousseau, M.C. (2011). *Proposal: A mixed methods appraisal tool for systematic mixed studies reviews.* Retrieved on [date] from http://mixedmethodsappraisaltoolpublic.pbworks.com. Archived by WebCite® at http://www.webcitation.org/5tTRTc9yJ
RCT	Cochrane Risk of Bias (RoB 2)	https://methods.cochrane.org/risk-bias-2
Cohort Study	The Newcastle-Ottawa Scale	https://www.ohri.ca/programs/clinical_epidemiology/oxford.asp
Case control Study	The Newcastle-Ottawa Scale	https://www.ohri.ca/programs/clinical_epidemiology/oxford.asp
Retrospective observational	Adapted Quality Assessment Tool for Retrospective and prospective observational studies	See the following supplementary material
Prospective observational	Adapted Quality Assessment Tool for Retrospective and prospective observational studies	See the following supplementary material

3.7. Data extraction

A set of variables will be defined and relevant information will be extracted from the included records. Should it not be possible to extract meaningful data from an included study, this will be excluded and tagged as "non-extractable data" in the list of excluded full-text records.

The included references will be summarised by collecting relevant information in a standardised Excel format, per study. Where possible, a pre-defined list of options per variable will be created (e.g. on study design, aim of the technology, etc.). The unit for data extraction will be study, instead of article. A study is defined as a screening approach and/or follow up for a defined population group, in a defined country, over a discrete period of time. According to this definition, a single study may be presented in more than one article. Whether both articles or just one (and in that case, which) article will be selected for data extraction will be decided on a case-by-case basis.

The Table 4 reports the information extracted in the Excel sheet.

Table S4. Data Extraction form.

Variable	Definition	Type of variable
**PMID**	PubMed ID	*Number*
**Title**	Article title	*Free text*
**Journal**	Journal of publication	*Free text*
**Year of publication**	Year of publication of the article	*Number*
**Study design**	Type of the study (as stated by the author)	*Prospective observational, Retrospective observational, Case-control, Cohort, Quasi-experimental, RCT*
**Country**	Country of the study	*Free text*
**Period of study**	Study period when the study was conducted	*Free text*
**Duration (Months)**	Duration of study	*Number*
**Last year of the study**	Last year of the study	*Number*
**Single/ Multicentre**	Single: study conducted in one hospital/LCTF; Multicenter: more than one hospital/LCTF	*Single, Multicentre*
**Comparative study**	Does the study compare the new technology with something else?	*Yes, No, Unclear*
**Study aims/objectives**	Aims/objectives of the study	*Free text*
**Setting of study**	Study setting (hospital and/or LCTF)	*General Hospital, LCTF, University, Tertiary non-university, Other*
**Other (specific) - ward**	Ward where was conducted by the study	*ICU, Renal transplant, Liver transplant, Maternity ward, All, More than one, Other, Haematology, Burn unit, Orthopedy, Geriatric, Oncology, Coronary care unit, ER, Internal medicine, Pediatric*
**Digital technology classification**	Type of technology investigated	*Robotics, Blockchain, Cloud computing, Artificial Intelligence (AI), Machine Learning, Natural Language processing, Big data analytics, Health informatics, Digital health/e-health/m-*

		health, Electronic health records (EHRs), Virtual reality, Smartphone and tablet computing devices, Internet of things (IoT), Wearables
**Concise description of digital technology**	Short description of the digital technology (when/if described by the authors)	*Free text*
**Aim of technology**	Purpose of the technology under study	*Surveillance, Outbreak detection*
**Intervention under surveillance**	Type of intervention (surgical site infection control, follow-up after discharge, etc.) on which the new tool was applied	*Free text*
**Infectious disease under investigation**	Infectious disease targeted by digital technology	*Free text*
**Potential benefits**	Potential benefits of the technology for public health functions	*Free text*
**Potential negative impacts**	Potential negative aspects for public health functions	*Free text*
**Obstacles**	Obstacles/barriers to implementation	*Free text*
**Comments**	Additional comments	*Free text*
**Target population**	Target population of the study	*Number*
**Sex (M)**	Number of male subjects	*Number*
**Age**	Age (median or average)	*Number (Median or Mean)*
**Comparative population**	Control population	*Number*
**Sex (M)**	Number of male subjects in the control population	*Number*
**Age**	Age (median or mean) of control population	*Number (Median or Mean)*
**Pros and cons for the new technologies vs old one**	What do the authors say are the pros or cons of the new method used compared to the previous traditional one	*Free text*
**Quality assessment tool**	Used quality assessment tools	*Cochrane Risk of Bias (RoB 2), The Newcastle-Ottawa Scale, Mixed Methods Appraisal Tool (MMAT) – Version 2011, Critical Appraisal Skills Programme (CASP), Adapted Quality*

		Assessment Tool for Retrospective and prospective observational studies
**Quality value**	Quality value (result of the quality assessment tool)	*Free text*

3.8. Data synthesis

The extracted data will be analysed, summarised sorted by research question. Data on innovative approaches will be presented by country, setting ward and aim of the new technology. If appropriate, data will also be analysed comparing traditional and innovative techniques for surveillance. Data will be reported for qualitative and quantitative analysis.

4. Manuscript writing

The findings from the project will be reported in a scientific manuscript to be published in an international peer-reviewed journal. Authorship will be attributed according to ICMJE recommendations https://www.icmje.org

Table S5. Search string

**PubMed**	
**Concept 1**	**Healthcare Associated Infection**
(Cross Infection[MeSH Terms] OR "Cross Infection"[Title/Abstract]) OR (urinar* OR "urinary tract" OR ("Surgical site" AND infection*) OR sepsis OR "Healthcare-Associated Pneumonia"[Title/Abstract]) OR (Bacteriuria OR Pyuria OR "Catheter-Related Infections" OR "Surgical Wound Dehiscence" OR "Surgical Wound Infection*" OR "Prosthesis-Related Infection*"[Title/Abstract]) OR ("Surgical Wound Infection"[MeSH Terms] OR "Puerperal Infection"[Title/Abstract]) OR (enterobacteriaceae OR enterobacterales OR citrobacter OR Enterobacter* OR escherichia OR hafnia OR klebsiella OR morganell* OR proteus OR providencia OR serratia OR "e coli" OR "e.coli" OR Citrobacter[MeSH Terms] OR Escherichia[MeSH Terms] OR Hafnia[MeSH Terms] OR Morganella[MeSH Terms] OR Proteus[MeSH Terms] OR Providencia[MeSH Terms] OR	1,503,191 results

Serratia[MeSH Terms] OR "E.aerogenes" OR "e aerogenes" OR "k.oxytoca" OR "k oxytoca" OR "k pneumonia*" OR "k.pneumonia*" OR "e cloacae" OR "e.cloacae" OR Enterobacter[MeSH Terms] OR Klebsiella[MeSH Terms][Title/Abstract]) OR (Acinetobacter[MeSH Terms] OR "acinetobacter baumannii"[Title/Abstract]) OR ("pseudomonas aeruginosa"[MeSH Terms] OR (pseudomonas AND aeruginosa) OR "pseudomonas aeruginosa" OR Stenotrophomonas[Title/Abstract]) OR (Candida[MeSH Terms]) OR ("Vancomycin-Resistant Enterococci" OR VRE[Title/Abstract]) OR ("Staphilococcus aureus" OR MRSA OR "Methicillin-Resistant Staphylococcus Aureus"[Title/Abstract])	
**Concept 2**	**Setting**
((ward*[Title/Abstract] OR hospital*[Title/Abstract] OR unit*[Title/Abstract] OR ICU[Title/Abstract] OR ICU [MeSH Terms] OR HDU*[Title/Abstract] OR PICU*[Title/Abstract] OR SCBU*[Title/Abstract] OR CCU*[Title/Abstract] OR NICU*[Title/Abstract] OR ITU*[Title/Abstract] OR er*[Title/Abstract] OR "emergency room"[Title/Abstract] OR "emergency department"[Title/Abstract] OR "casualty department"[Title/Abstract] OR clinic*[Title/Abstract] OR facility*[Title/Abstract]) OR "Academic Medical Cent*"[Title/Abstract] OR "Teaching Hospital*"[Title/Abstract] OR "Birthing Centers"[Title/Abstract] OR "Health Facility Environment"[Title/Abstract] OR "Hospital Units"[Title/Abstract] OR "Community Hospital*"[Title/Abstract] OR "General Hospital*"[Title/Abstract] OR "Group Practice Hospital*"[Title/Abstract] OR "High-Volume Hospital*"[Title/Abstract] OR "Low-Volume Hospital*"[Title/Abstract] OR "Private Hospital*"[Title/Abstract] OR "Public Hospital*"[Title/Abstract] OR "Rural Hospital*"[Title/Abstract] OR "Satellite	7,589,641 results

Hospital*"[Title/Abstract] OR "Special Hospital*"[Title/Abstract] OR "Teaching Hospital*"[Title/Abstract] OR "Urban Hospital*"[Title/Abstract] OR "Secondary Care Center*"[Title/Abstract] OR "Tertiary Care Center*"[Title/Abstract] OR "Nursing Home*"[Title/Abstract] OR "Intermediate Care Facilit*"[Title/Abstract] OR "Skilled Nursing Facilit*"[Title/Abstract] OR "Residential Facilit*"[Title/Abstract] OR "Assisted Living Facilit*"[Title/Abstract] OR "Group Home*"[Title/Abstract] OR "Homes for the Aged"[Title/Abstract] OR hospitals[MeSH Terms] OR Hospital Units [MeSH Terms] OR Rehabilitation Centers [MeSH Terms] OR Academic Medical Centers [MeSH Terms] OR Residential Facilities [MeSH Terms] OR Nursing Homes [MeSH Terms])	
**Concept 3**	**Surveillance**
((screen*[Title/Abstract] OR surveill*[Title/Abstract] OR monitor*[Title/Abstract] OR control[Title/Abstract]) AND (carriage*[Title/Abstract] OR coloni*[Title/Abstract] OR infect*[Title/Abstract]))	537,576 results
**Concept 4**	**Innovation**
(Algorithm*[MeSH Terms] OR Algorithm[Title/Abstract])<br>OR<br>(digital technolog*[tw] OR "information technology"[MeSH Terms] OR information technolog*[tw] OR communication technolog*[tw] OR "ICT"[tw] OR new technolog*[tw] OR digital innovation*[tw] OR emerging technolog*[tw] OR machine learning[tw] OR blockchain[tw] OR "data mining"[tw] OR datamining[tw] OR automation[tw] OR "augmented reality"[tw] OR "virtual reality"[tw] OR virtual setting*[tw] OR cloud[tw] OR "internet of things"[tw] OR "iot"[tw] OR 3G[tw] OR 4G[tw] OR 5G[tw] OR "artificial intelligence"[tw] OR "ai"[tw] OR "big data"[tw] OR "deep learning"[tw] OR "nano"[tw] OR "digital health"[tw] OR	1,028,774 results

robotic*[tw] OR quantum comput*[tw] OR "additive manufacturing"[tw] OR ((cellular phone*[tw] OR cell phone*[tw] OR mobile phone*[tw]) AND (health technol*[tw] OR biomedical technol*[tw] OR medical technol*[tw])) OR remote sensing technol*[tw] OR smart fabric*[tw] OR wearables[tw] OR wearable technol*[tw] OR wearable electronic device*[tw] OR "Data Mining"[MeSH Terms] OR "Automation"[MeSH Terms] OR "Virtual Reality"[MeSH Terms] OR "Cloud Computing"[MeSH Terms] OR "Artificial Intelligence"[MeSH Terms] OR "Big Data"[MeSH Terms] OR ("Cell Phone"[MeSH Terms] AND "Biomedical Technology"[MeSH Terms]) OR "Remote Sensing Technology"[MeSH Terms] OR "Wearable Electronic Devices"[MeSH Terms])	
Concept 1 AND Concept 2 AND Concept 3 AND Concept 4	1,362 results
**Time restriction**	
From 20180101 to 20231027	644 results

**Scopus query**	
(TITLE-ABS-KEY ("Cross Infection" OR urinar* OR "urinary tract" OR ("Surgical site" AND infection*) OR sepsis OR "Healthcare-Associated Pneumonia" OR bacteriuria OR pyuria OR "Catheter-Related Infections" OR "Surgical Wound Dehiscence" OR "Surgical Wound Infection*" OR "Prosthesis-Related Infection*" OR "Surgical Wound Infection" OR "Puerperal Infection" OR enterobacteriaceae OR enterobacterales OR citrobacter OR enterobacter* OR escherichia OR hafnia OR klebsiella OR morganell* OR proteus OR providencia OR serratia OR "e coli" OR "e.coli" OR "E.aerogenes" OR "e aerogenes" OR "k.oxytoca" OR "k oxytoca" OR "k pneumonia*" OR "k.pneumonia*" OR "e cloacae" OR "e.cloacae" OR acinetobacter	368 results

OR "acinetobacter baumannii" OR "pseudomonas aeruginosa" OR (pseudomonas AND aeruginosa) OR "pseudomonas aeruginosa" OR stenotrophomonas OR candida OR "Vancomycin-Resistant Enterococci" OR vre OR "Staphilococcus aureus" OR mrsa OR "Methicillin-Resistant Staphylococcus Aureus")) AND (TITLE-ABS-KEY ((carriage* OR coloni* OR infect*))) AND (TITLE-ABS-KEY ((screen* OR surveill* OR monitor* OR control))) AND (TITLE-ABS-KEY ((ward* OR hospital* OR unit* OR icu OR hdu* OR picu* OR scbu* OR ccu* OR nicu* OR itu* OR er* OR "emergency room" OR "emergency department" OR "casualty department" OR clinic* OR facility* OR "Academic Medical Cent*" OR "Teaching Hospital*" OR "Birthing Centers" OR "Health Facility Environment" OR "Hospital Units" OR "Community Hospital*" OR "General Hospital*" OR "Group Practice Hospital*" OR "High-Volume Hospital*" OR "Low-Volume Hospital*" OR "Private Hospital*" OR "Public Hospital*" OR "Rural Hospital*" OR "Satellite Hospital*" OR "Special Hospital*" OR "Teaching Hospital*" OR "Urban Hospital*" OR "Secondary Care Center*" OR "Tertiary Care Center*" OR "Rehabilitation Cent*" OR "Residential Facilit*" OR "Assisted Living Facilit*" OR "Group Home*" OR "Homes for the Aged" OR "Nursing Home*" OR "Intermediate Care Facilit*" OR "Skilled Nursing Facilit*"))) AND (ALL ((algorithm OR digital AND technolog* OR "information technolog*" OR communication AND technolog* OR "ICT" OR new AND technolog* OR digital AND innovation* OR emerging AND technolog* OR "machine learning" OR blockchain OR "data mining" OR datamining OR automation OR "augmented reality" OR "virtual reality" OR virtual AND setting* OR cloud OR "internet of things" OR "iot" OR 3g OR 4g OR 5g OR "artificial intelligence" OR "ai" OR "big data" OR "deep learning" OR "nano" OR "digital health" OR robotic*	

OR "quantum comput*" OR "additive manufacturing" OR (cellular AND phone* OR "cell phone*" OR "mobile phone*") AND (health AND technol* OR biomedical AND technol* OR medical AND technol*) OR "remote sensing technol*" OR "smart fabric*" OR wearables OR wearable AND technol* OR "wearable electronic device*"))) AND PUBYEAR > 2017 AND PUBYEAR < 2025	

**Web of Science query**	
**Concept 1**	**Healthcare associated infection**
AB=(("Cross Infection" OR urinar* OR "urinary tract" OR ("Surgical site" AND infection*) OR sepsis OR "Healthcare-Associated Pneumonia" OR bacteriuria OR pyuria OR "Catheter-Related Infections" OR "Surgical Wound Dehiscence" OR "Surgical Wound Infection*" OR "Prosthesis-Related Infection*" OR "Surgical Wound Infection" OR "Puerperal Infection" OR enterobacteriaceae OR enterobacterales OR citrobacter OR enterobacter* OR escherichia OR hafnia OR klebsiella OR morganell* OR proteus OR providencia OR serratia OR "e coli" OR "e.coli" OR "E.aerogenes" OR "e aerogenes" OR "k.oxytoca" OR "k oxytoca" OR "k pneumonia*" OR "k.pneumonia*" OR "e cloacae" OR "e.cloacae" OR acinetobacter OR "acinetobacter baumannii" OR "pseudomonas aeruginosa" OR (pseudomonas AND aeruginosa) OR "pseudomonas aeruginosa" OR stenotrophomonas OR candida OR "Vancomycin-Resistant Enterococci" OR vre OR "Staphilococcus aureus" OR mrsa OR "Methicillin-Resistant Staphylococcus Aureus"))	814,463 results
**Concept 2**	**Setting**
AB=((ward* OR hospital* OR unit* OR icu OR hdu* OR picu* OR scbu* OR ccu* OR nicu* OR itu* OR er* OR "emergency room" OR "emergency department" OR "casualty department" OR clinic* OR facility* OR “Academic Medical Cent*” OR “Teaching	9,201,432 results

Hospital*” OR “Birthing Centers” OR “Health Facility Environment” OR “Hospital Units” OR “Community Hospital*” OR “General Hospital*” OR “Group Practice Hospital*” OR “High-Volume Hospital*” OR “Low-Volume Hospital*” OR “Private Hospital*” OR “Public Hospital*” OR “Rural Hospital*” OR “Satellite Hospital*” OR ”Special Hospital*” OR “Teaching Hospital*” OR “Urban Hospital*” OR “Secondary Care Center*” OR “Tertiary Care Center*” OR “Rehabilitation Cent*” OR “Residential Facilit*” OR “Assisted Living Facilit*” OR “Group Home*” OR “Homes for the Aged” OR “Nursing Home*” OR “Intermediate Care Facilit*” OR “Skilled Nursing Facilit*”))	
**Concept 3**	**Surveillance**
AB=((screen* OR surveill* OR monitor* OR control)) AND ((carriage* OR coloni* OR infect*))	580,802 results
**Concept 4**	**Innovation**
AB=((Algorithm OR digital technolog* OR "information technolog*" OR communication technolog* OR "ICT" OR new technolog* OR digital innovation* OR emerging technolog* OR "machine learning" OR blockchain OR "data mining" OR datamining OR automation OR "augmented reality" OR "virtual reality" OR virtual setting* OR cloud OR "internet of things" OR "iot" OR 3G OR 4G OR 5G OR "artificial intelligence" OR "ai" OR "big data" OR "deep learning" OR "nano" OR "digital health" OR robotic* OR "quantum comput*" OR "additive manufacturing" OR (cellular phone* OR "cell phone*" OR "mobile phone*") AND (health technol* OR biomedical technol* OR medical technol*) OR "remote sensing technol*" OR "smart fabric*" OR wearables OR wearable technol* OR "wearable electronic device*"))	4,343,958 results
#5 AND #4 AND #3 AND #2 AND #1 and 2023 or 2022 or 2021 or 2020 or 2019 or 2018 (Publication Years)	651 results

Cochrane Risk of Bias Tool for Randomized Controlled Trials

**RANDOM SEQUENCE GENERATION**<br>**Selection bias (biased allocation to interventions) due to inadequate generation of a randomised sequence.**	
Criteria for a judgment of 'Low risk' of bias.	The investigators describe a random component in the sequence generation process such as:<br>• Referring to a random number table;<br>• Using a computer random number generator;<br>• Coin tossing;<br>• Shuffling cards or envelopes;<br>• Throwing dice;<br>• Drawing of lots;<br>• Minimization*.<br>*Minimization may be implemented without a random element, and this is considered to be equivalent to being random.
Criteria for the judgment of 'High risk' of bias.	The investigators describe a non-random component in the sequence generation process. Usually, the description would involve some systematic, non-random approach, for example:<br>• Sequence generated by odd or even date of birth;<br>• Sequence generated by some rule based on date (or day) of admission;<br>• Sequence generated by some rule based on hospital or clinic record number.<br>Other non-random approaches happen much less frequently than the systematic approaches mentioned above and tend to be obvious. They usually involve judgement or some method of non-random categorization of participants, for example:<br>• Allocation by judgement of the clinician;<br>• Allocation by preference of the participant;<br>• Allocation based on the results of a laboratory test or a series of tests;<br>• Allocation by availability of the intervention.
Criteria for the judgment of 'Unclear risk' of bias.	Insufficient information about the sequence generation process to permit judgement of 'Low risk' or 'High risk'.

ALLOCATION CONCEALMENT<br>**Selection bias (biased allocation to interventions) due to inadequate concealment of allocations prior to assignment.**	
Criteria for a judgment of 'Low risk' of bias.	Participants and investigators enrolling participants could not foresee assignment because one of the following, or an equivalent method, was used to conceal allocation:<br>• Central allocation (including telephone, web-based and pharmacy-controlled randomization);<br>• Sequentially numbered drug containers of identical appearance;<br>• Sequentially numbered, opaque, sealed envelopes.
Criteria for the judgment of 'High risk' of bias.	Participants or investigators enrolling participants could possibly foresee assignments and thus introduce selection bias, such as allocation based on:<br>• Using an open random allocation schedule (e.g. a list of random numbers);<br>• Assignment envelopes were used without appropriate safeguards (e.g. if envelopes were unsealed or non-opaque or not sequentially numbered);<br>• Alternation or rotation;<br>• Date of birth;<br>• Case record number;<br>• Any other explicitly unconcealed procedure.
Criteria for the judgment of 'Unclear risk' of bias.	Insufficient information to permit judgement of 'Low risk' or 'High risk'. This is usually the case if the method of concealment is not described or not described in sufficient detail to allow a definite judgement – for example if the use of assignment envelopes is described, but it remains unclear whether envelopes were sequentially numbered, opaque and sealed.
**SELECTIVE REPORTING**<br>**Reporting bias due to selective outcome reporting.**	
Criteria for a judgment of 'Low risk' of bias.	Any of the following:<br>• The study protocol is available and all of the study's pre-specified (primary and secondary) outcomes that are of interest in the review have been reported in the pre-specified way;<br>• The study protocol is not available but it is clear that the published reports include all expected outcomes, including those that were pre-specified (convincing text of this nature may be uncommon).
Criteria for the judgment of 'High risk' of bias.	Any one of the following:<br>• Not all of the study's pre-specified primary outcomes have been reported;<br>• One or more primary outcomes is reported using measurements, analysis methods or subsets of the data (e.g. subscales) that were not pre-specified;<br>• One or more reported primary outcomes were not pre-specified

	(unless clear justification for their reporting is provided, such as an unexpected adverse effect);<br>• One or more outcomes of interest in the review are reported incompletely so that they cannot be entered in a meta-analysis;<br>• The study report fails to include results for a key outcome that would be expected to have been reported for such a study.
Criteria for the judgment of 'Unclear risk' of bias.	Insufficient information to permit judgement of 'Low risk' or 'High risk'. It is likely that the majority of studies will fall into this category.

OTHER BIAS<br>**Bias due to problems not covered elsewhere in the table.**	
Criteria for a judgment of 'Low risk' of bias.	The study appears to be free of other sources of bias.
Criteria for the judgment of 'High risk' of bias.	There is at least one important risk of bias. For example, the study:<br>• Had a potential source of bias related to the specific study design used; or<br>• Has been claimed to have been fraudulent; or<br>• Had some other problem.
Criteria for the judgment of 'Unclear risk' of bias.	There may be a risk of bias, but there is either:<br>• Insufficient information to assess whether an important risk of bias exists; or<br>• Insufficient rationale or evidence that an identified problem will introduce bias.
**BLINDING OF PARTICIPANTS AND PERSONNEL**<br>**Performance bias due to knowledge of the allocated interventions by participants and personnel during the study.**	
Criteria for a judgment of 'Low risk' of bias.	Any one of the following:<br>• No blinding or incomplete blinding, but the review authors judge that the outcome is not likely to be influenced by lack of blinding;<br>• Blinding of participants and key study personnel ensured, and unlikely that the blinding could have been broken.
Criteria for the judgment of 'High risk' of bias.	Any one of the following:<br>• No blinding or incomplete blinding, and the outcome is likely to be influenced by lack of blinding;<br>• Blinding of key study participants and personnel attempted, but likely that the blinding could have been broken, and the outcome is likely to be influenced by lack of blinding.
Criteria for the judgment of 'Unclear risk' of bias.	Any one of the following:<br>• Insufficient information to permit judgment of 'Low risk' or 'High risk';<br>• The study did not address this outcome.

BLINDING OF OUTCOME ASSESSMENT<br>**Detection bias due to knowledge of the allocated interventions by outcome assessors.**	
Criteria for a judgment of 'Low risk' of bias.	Any one of the following:<br>• No blinding of outcome assessment, but the review authors judge that the outcome measurement is not likely to be influenced by lack of blinding;<br>• Blinding of outcome assessment ensured, and unlikely that the blinding could have been broken.
Criteria for the judgment of 'High risk' of bias.	Any one of the following:<br>• No blinding of outcome assessment, and the outcome measurement is likely to be influenced by lack of blinding;<br>• Blinding of outcome assessment, but likely that the blinding could have been broken, and the outcome measurement is likely to be influenced by lack of blinding.
Criteria for the judgment of 'Unclear risk' of bias.	Any one of the following:<br>• Insufficient information to permit judgment of 'Low risk' or 'High risk';<br>• The study did not address this outcome.
**INCOMPLETE OUTCOME DATA**<br>**Attrition bias due to amount, nature or handling of incomplete outcome data.**	
Criteria for a judgment of 'Low risk' of bias.	Any one of the following:<br>• No missing outcome data;<br>• Reasons for missing outcome data unlikely to be related to true outcome (for survival data, censoring unlikely to be introducing bias);<br>• Missing outcome data balanced in numbers across intervention groups, with similar reasons for missing data across groups;<br>• For dichotomous outcome data, the proportion of missing outcomes compared with observed event risk not enough to have a clinically relevant impact on the intervention effect estimate;<br>• For continuous outcome data, plausible effect size (difference in means or standardized difference in means) among missing outcomes not enough to have a clinically relevant impact on observed effect size;<br>• Missing data have been imputed using appropriate methods.
Criteria for the judgment of 'High risk' of bias.	Any one of the following:<br>• Reason for missing outcome data likely to be related to true outcome, with either imbalance in numbers or reasons for missing data across intervention groups;<br>• For dichotomous outcome data, the proportion of missing outcomes compared with observed event risk enough to induce clinically relevant bias in intervention effect estimate;

	• For continuous outcome data, plausible effect size (difference in means or standardized difference in means) among missing outcomes enough to induce clinically relevant bias in observed effect size;<br>• 'As-treated' analysis done with substantial departure of the intervention received from that assigned at randomization;<br>• Potentially inappropriate application of simple imputation.
Criteria for the judgment of 'Unclear risk' of bias.	Any one of the following:<br>• Insufficient reporting of attrition/exclusions to permit judgement of 'Low risk' or 'High risk' (e.g. number randomized not stated, no reasons for missing data provided);<br>• The study did not address this outcome.

Thresholds for Converting the Cochrane Risk of Bias Tool to AHRQ Standards (Good, Fair, and Poor)

Good quality: All criteria met (i.e. low for each domain)

Using the Cochrane ROB tool, it is possible for a criterion to be met even when the element was technically not part of the method. For instance, a judgment that knowledge of the allocated interventions was adequately prevented can be made even if the study was not blinded, if EPC team members judge that the outcome and the outcome measurement are not likely to be influenced by lack of blinding.

Fair quality: One criterion not met (i.e. high risk of bias for one domain) or two criteria unclear, and the assessment that this was **unlikely** to have biased the outcome, and there is no known important limitation that could invalidate the results

Poor quality: One criterion not met (i.e. high risk of bias for one domain) or two criteria unclear, and the assessment that this was **likely** to have biased the outcome, and there are important limitations that could invalidate the results

Poor quality: Two or more criteria listed as high or unclear risk of bias

Cochrane Risk of Bias Tool

Use the modified Cochrane Collaboration tool to assess risk of bias for randomized controlled trials. Bias is assessed as a judgment (high, low, or unclear) for individual elements from five domains (selection, performance, attrition, reporting, and other).

AUB KQ1 Risk of Bias Assessment (Reference ID #)

Domain	Description	High Risk of Bias	Low Risk of Bias	Unclear Risk of Bias	Reviewer Assessment	Reviewer Comments
Selection bias ***Random sequence generation***	Described the method used to generate the allocation sequence in sufficient detail to allow an assessment of whether it should produce comparable groups	Selection bias (biased allocation to interventions) due to inadequate generation of a randomized sequence	Random sequence generation method should produce comparable groups	Not described in sufficient detail	**High**<br>**Low**<br>**Unclear**	
Selection bias ***Allocation concealment***	Described the method used to conceal the allocation sequence in sufficient detail to determine whether intervention allocations could have been foreseen before or during enrollment	Selection bias (biased allocation to interventions) due to inadequate concealment of allocations prior to assignment	Intervention allocations likely could not have been foreseen in before or during enrollment	Not described in sufficient detail	**High**<br>**Low**<br>**Unclear**	
Reporting bias ***Selective reporting***	Stated how the possibility of selective outcome reporting was examined by the authors and what was found	Reporting bias due to selective outcome reporting	Selective outcome reporting bias not detected	Insufficient information to permit judgment†	**High**<br>**Low**<br>**Unclear**	
Other bias ***Other sources of bias***	Any important concerns about bias not addressed above*	Bias due to problems not covered elsewhere in the table	No other bias detected	There may be a risk of bias, but there is either insufficient information to assess whether an important risk of bias exists or insufficient rationale or evidence that an identified problem will introduce bias	**High**<br>**Low**<br>**Unclear**	

* If particular questions/entries were pre-specified in the study's protocol, responses should be provided for each question/entry.
† It is likely that the majority of studies will fall into this category.

Assess each main or class of outcomes for each of the following. Indicate the specific outcome.

AUB KQ1 Risk of Bias Assessment (Reference ID #)

Outcome:

Domain	Description	High Risk of Bias	Low Risk of Bias	Unclear Risk of Bias	Reviewer Assessment	Reviewer Comments
Performance bias ***Blinding (participants and personnel)***	Described all measures used, if any, to blind study participants and personnel from knowledge of which intervention a participant received. Provided any information relating to whether the intended blinding was effective.	Performance bias due to knowledge of the allocated interventions by participants and personnel during the study.	Blinding was likely effective.	Not described in sufficient detail	**High**<br>**Low**<br>**Unclear**	
Detection bias ***Blinding (outcome assessment)***	Described all measures used, if any, to blind outcome assessors from knowledge of which intervention a participant received. Provided any information relating to whether the intended blinding was effective.	Detection bias due to knowledge of the allocated interventions by outcome assessors.	Blinding was likely effective.	Not described in sufficient detail	**High**<br>**Low**<br>**Unclear**	
Attrition bias ***Incomplete outcome data***	Described the completeness of outcome data for each main outcome, including attrition and exclusions from the analysis. Stated whether attrition and exclusions were reported, the numbers in each intervention group (compared with total randomized participants), reasons for attrition/exclusions where reported.	Attrition bias due to amount, nature or handling of incomplete outcome data.	Handling of incomplete outcome data was complete and unlikely to have produced bias	Insufficient reporting of attrition/exclusions to permit judgment (e.g., number randomized not stated, no reasons for missing data provided)	**High**<br>**Low**<br>**Unclear**	

NEWCASTLE - OTTAWA QUALITY ASSESSMENT SCALE
CASE CONTROL STUDIES

Note: A study can be awarded a maximum of one star for each numbered item within the Selection and Exposure categories. A maximum of two stars can be given for Comparability.

Selection

1) Is the case definition adequate?
   a) yes, with independent validation ❋
   b) yes, eg record linkage or based on self reports
   c) no description

2) Representativeness of the cases
   a) consecutive or obviously representative series of cases ❋
   b) potential for selection biases or not stated

3) Selection of Controls
   a) community controls ❋
   b) hospital controls
   c) no description

4) Definition of Controls
   a) no history of disease (endpoint) ❋
   b) no description of source

Comparability

1) Comparability of cases and controls on the basis of the design or analysis
   a) study controls for________________(Select the most important factor.) ❋
   b) study controls for any additional factor ❋ (This criteria could be modified to indicate specific control for a second important factor.)

Exposure

1) Ascertainment of exposure
   a) secure record (eg surgical records) ❋
   b) structured interview where blind to case/control status ❋
   c) interview not blinded to case/control status
   d) written self report or medical record only
   e) no description

2) Same method of ascertainment for cases and controls
   a) yes ❋
   b) no

3) Non-Response rate
   a) same rate for both groups ❋
   b) non respondents described
   c) rate different and no designation

NEWCASTLE - OTTAWA QUALITY ASSESSMENT SCALE
COHORT STUDIES

Note: A study can be awarded a maximum of one star for each numbered item within the Selection and Outcome categories. A maximum of two stars can be given for Comparability

Selection

1) Representativeness of the exposed cohort
   a) truly representative of the average _______________ (describe) in the community ❋
   b) somewhat representative of the average ______________ in the community ❋
   c) selected group of users eg nurses, volunteers
   d) no description of the derivation of the cohort

2) Selection of the non exposed cohort
   a) drawn from the same community as the exposed cohort ❋
   b) drawn from a different source
   c) no description of the derivation of the non exposed cohort

3) Ascertainment of exposure
   a) secure record (eg surgical records) ❋
   b) structured interview ❋
   c) written self report
   d) no description

4) Demonstration that outcome of interest was not present at start of study
   a) yes ❋
   b) no

Comparability

1) Comparability of cohorts on the basis of the design or analysis
   a) study controls for _____________ (select the most important factor) ❋
   b) study controls for any additional factor ❋ (This criteria could be modified to indicate specific control for a second important factor.)

Outcome

1) Assessment of outcome
   a) independent blind assessment ❋
   b) record linkage ❋
   c) self report
   d) no description

2) Was follow-up long enough for outcomes to occur
   a) yes (select an adequate follow up period for outcome of interest) ❋
   b) no

3) Adequacy of follow up of cohorts
   a) complete follow up - all subjects accounted for ❋
   b) subjects lost to follow up unlikely to introduce bias - small number lost - >_____% (select an adequate %) follow up, or description provided of those lost) ❋
   c) follow up rate <_____% (select an adequate %) and no description of those lost
   d) no statement

Thresholds for converting the Newcastle-Ottawa scales to AHRQ standards (good, fair, and poor):
Good quality: 3 or 4 stars in selection domain AND 1 or 2 stars in comparability domain AND 2 or 3 stars in outcome/exposure domain
Fair quality: 2 stars in selection domain AND 1 or 2 stars in comparability domain AND 2 or 3 stars in outcome/ exposure domain
Poor quality: 0 or 1 star in selection domain OR 0 stars in comparability domain OR 0 or 1 stars in outcome/ exposure domain

Adapted Quality Assessment Tool for Before and After Studies

Criteria	Yes	No	Other (CD, NR, NA)*
1. Was the study question or objective clearly stated?			
2. Were eligibility/selection criteria for the study population prespecified and clearly described?			
3. Were the participants in the study representative of those who would be eligible for the test/service/intervention in the general or clinical population of interest?			
4. Were all eligible participants that met the prespecified entry criteria enrolled?			
5. Was the sample size sufficiently large to provide confidence in the findings?			
6. Was the test/service/intervention clearly described and delivered consistently across the study population?			
7. Were the outcome measures prespecified, clearly defined, valid, reliable, and assessed consistently across all study participants?			
8. Was the loss to follow-up after baseline 20% or less?			
9. Was the statistical approach used to analyze the data clearly described and appropriate?			

Criteria	Yes	No	Other (CD, NR, NA)*
10. Were the basic data results adequately described?			
11. Were the results presented for all the analyses described in the methods?			

*CD, cannot determine; NA, not applicable; NR, not reported

Number of "Yes" Answer / Total elements

If the study does not present any data regarding the acquisition or progression rates of colonization to infection, we may not answer question 8 by excluding it from the evaluation.

Number of "Yes" Answers	Total elements	Quality rate
11	11	High Quality
10	11	High Quality
9	11	High Quality
8	11	Low Quality
7	11	Low Quality
6	11	Low Quality
5	11	Low Quality
4	11	Very Low Quality
3	11	Very Low Quality
2	11	Very Low Quality
1	11	Very Low Quality
0	11	Very Low Quality

Number of "Yes" Answers	Total elements	Quality rate
10	10	High Quality
9	10	High Quality
8	10	High Quality
7	10	Low Quality
6	10	Low Quality
5	10	Low Quality
4	10	Low Quality
3	10	Very Low Quality
2	10	Very Low Quality
1	10	Very Low Quality
0	10	Very Low Quality

Articles excluded in full text

PMID	Title	Authors	Year	DOI	Inclusion FT	Aim of exclusion
31893042	Molecular surveillance of carbapenemase-producing Pseudomonas aeruginosa at three medical centres in Cologne, Germany	Schäfer E, Malecki M, Tellez-Castillo	2019	10.1186/s13756-019-0665-5	No	No new technologies
126	Application of intelligent nursing based on cloud computing of internet of things in children with pneumonia and sepsis treated wit	Qin, AH; Liu, YL; Shao, CM; Dong, HY	2023	10.1177/1721727X231194144	No	Outcome
28807836	Development and evaluation of the automated risk assessment system for multidrug-resistant organisms (autoRAS-MDRO)	Hur EY, Jin YJ, Jin TX, Lee SM.	2018	10.1016/j.jhin.2017.08.004	No	No new technologies
29439746	Antimicrobial Stewardship Program Implementation of a Quality Improvement Intervention Using Real-Time Feedback and an Elect	Rosa R, Zavala B, Cain N, Anjan S, Ar	2018	10.1017/ice.2017.325	No	Outcome
38	Machine Learning Algorithms to Predict Healthcare Associated Infections in a Neonatal Intensive Care Unit	Montella E.; Marino M.R.; Scala A.; T	2023	10.1007/978-3-031-25191-7_38	No	Outcome
34184637	Rapid feedback on hospital onset SARS-CoV-2 infections combining epidemiological and sequencing data	Stirrup O, Hughes J, Parker M, Partri	2021	10.7554/eLife.65828	No	Outcome
37369173	Development and clinical impact assessment of a machine-learning model for early prediction of late-onset sepsis	van den Berg MAM, Medina OOAG,	2023	10.1016/j.compbiomed.2023.107156	No	Outcome
35700927	Reducing CAUTI in patients with acute urinary retention in the critical care setting: A pilot study with electronic medical record ana	Lilley T, Teixeira-Poit S, Wenner J, Pr	2023	10.1016/j.ajic.2022.06.005	No	Outcome
29366555	Feasibility of an Image-Based Mobile Health Protocol for Postoperative Wound Monitoring	Gunter, RL; Fernandes-Taylor, S; Rah	2018	10.1016/j.jamcollsurg.2017.12.013	No	Type of study
35249794	Healthcare-associated infections in adult intensive care unit patients: Changes in epidemiology, diagnosis, prevention and contribu	Blot S, Ruppé E, Harbarth S, Asehnou	2022	10.1016/j.iccn.2022.103227	No	Type of study
94	Systematic development of an mHealth app to prevent healthcare-associated infections by involving patients: 'Participatient'	Bentvelsen R.G.; van der Vaart R.; Ve	2021	10.1016/j.ceh.2021.03.001	No	Type of study
34496983	A computerized indicator for surgical site infection (SSI) assessment after total hip or total knee replacement: The French ISO-ORTH	Grammatico-Guillon L, Miliani K, Bar	2022	10.1017/ice.2021.371	No	No extractable data
37800568	Prediction models of surgical site infection after gastrointestinal surgery: a nationwide prospective cohort study	Yang Y, Zhang X, Zhang J, Zhu J, Wan	2023	10.1097/JS9.0000000000000808	No	No extractable data
34745489	Susceptible-Infected-Removed Mathematical Model under Deep Learning in Hospital Infection Control of Novel Coronavirus Pneum	Liu T, Bai Y, Du M, Gao Y, Liu Y.	2021	10.1155/2021/1535046	No	Type of study
37386032	Influenza transmissibility among patients and health-care professionals in a geriatric short-stay unit using individual contact data	Gustin M.-P.; Pujo-Menjouet L.; Van	2023	10.1038/s41598-023-36908-5	No	Outcome
36618079	Machine learning-assisted ensemble analysis for the prediction of urinary tract infection in elderly patients with ovarian cancer afte	Ai J, Hu Y, Zhou FF, Liao YX, Yang T.	2022	10.5306/wjco.v13.i12.967	No	Outcome
33711939	A spatiotemporal simulation study on the transmission of harmful microorganisms through connected healthcare workers in a hosp	van Niekerk J.M.; Stein A.; Doting M.	2021	10.1186/s12879-021-05954-7	No	Outcome
30069472	WMSS: A Web-Based Multitiered Surveillance System for Predicting CLABSI	Noaman AY, Ragab AHM, Al-Abdulla	2018	10.1155/2018/5419313	No	No extractable data
28482161	Gamification and Microlearning for Engagement With Quality Improvement (GAMEQI): A Bundled Digital Intervention for the Preve	Orwoll B.; Diane S.; Henry D.; Tsang	2018	10.1177/1062860617706542	No	Outcome
31525183	Fast and near-optimal monitoring for healthcare acquired infection outbreaks	Adhikari B, Lewis B, Vullikanti A, Jim	2019	10.1371/journal.pcbi.1007284	No	No extractable data
37753703	Preoperative Prediction of Postoperative Infections Using Machine Learning and Electronic Health Record Data	Zhuang Y, Dyas A, Meguid RA, Hend	2023	10.1097/SLA.0000000000006106	No	No extractable data
29486341	Predicting central line-associated bloodstream infections and mortality using supervised machine learning	Parreco JP, Hidalgo AE, Badilla AD, Il	2018	10.1016/j.jcrc.2018.02.010	No	No extractable data
29413730	Healthcare-associated ventriculitis and meningitis in a neuro-ICU: Incidence and risk factors selected by machine learning approach	Savin I, Ershova K, Kurdyumova N, E	2018	10.1016/j.jcrc.2018.01.022	No	Outcome
32371082	Prospective evaluation of an easy and reliable work flow for the screening of OXA-48-producing Klebsiella pneumoniae in endemic	Rodríguez-Lucas C, Rodicio MR, Rose	2020	10.1016/j.jhin.2020.04.044	No	Outcome
36961857	A novel, integrated approach for understanding and investigating Healthcare Associated Infections: A risk factors constellation anal	Carestia M, Andreoni M, Buonomo E	2023	10.1371/journal.pone.0282019	No	No new technologies
34725404	Probabilistic modelling of effects of antibiotics and calendar time on transmission of healthcare-associated infection	Laager, M; Cooper, B; Eyre, DW	2021	10.1038/s41598-021-00748-y	No	No new technologies
110	Optimal Decision of Dynamic Bed Allocation and Patient Admission with Buffer Wards during an Epidemic	Wang, CL; Yang, FF; Li, QL	2023	10.3390/math11030687	No	Outcome
35372245	Critical Care Database Comprising Patients With Infection	Xu P, Chen L, Zhu Y, Yu S, Chen R, Hu	2022	10.3389/fpubh.2022.852410	No	No new technologies
29061499	Reducing Inappropriate Testing for the Evaluation of Diarrhea Among Hospitalized Patients	Tewell C.E.; Talbot T.R.; Nelson G.E.;	2018	10.1016/j.amjmed.2017.10.006	No	Outcome
31525449	Use of health databases to deal with underreporting of surgical site infections due to suboptimal post-discharge follow-up	Gagliotti C, Buttazzi R, Ricciardi A, Ri	2020	10.1016/j.jhin.2019.09.009	No	No new technologies
35726554	Semiautomated surveillance of deep surgical site infections after colorectal surgeries: A multicenter external validation of two surv	Verberk JDM, van der Kooi TII, Heter	2023	10.1017/ice.2022.147	No	No new technologies
31884977	A framework to develop semiautomated surveillance of surgical site infections: An international multicenter study	van Rooden SM, Tacconelli E, Pujol M	2020	10.1017/ice.2019.321	No	No new technologies
33359550	National Infection Control Program in Turkey: The healthcare associated infection rate experiences over 10 years	Gozel, MG; Hekimoglu, CH; Gozel, EY	2021	10.1016/j.ajic.2020.12.013	No	No new technologies
31462548	Pilot Evaluation of a Fully Automated Bioinformatics System for Analysis of Methicillin-Resistant Staphylococcus aureus Genomes a	Brown NM, Blane B, Raven KE, Kuma	2019	10.1128/JCM.00858-19	No	Outcome
34039877	Modeling transmission of pathogens in healthcare settings	Stachel A, Keegan LT, Blumberg S; Cl	2021	10.1097/QCO.0000000000000742	No	Type of study
31761522	A Process Approach to Decreasing Hospital Onset Clostridium difficile Infections	Abbasi S, Singh F, Griffel M, Murphy	2020	10.1016/j.jcjq.2019.10.006	No	Outcome
37474597	Predicting sepsis onset using a machine learned causal probabilistic network algorithm based on electronic health records data	Valik JK, Ward L, Tanushi H, Johanss	2023	10.1038/s41598-023-38858-4	No	Outcome
36924997	The use of smart environments and robots for infection prevention control: A systematic literature review	Piaggio D, Zarro M, Pagliara S, Andel	2023	10.1016/j.ajic.2023.03.005	No	Outcome
37529839	Comorbidities directly extracted from the hospital database for adjusting SSI risk in the new national semiautomated surveillance s	Picard J, Nkoumazok B, Arnaud I, Ve	2023	10.1017/ice.2023.123	No	Outcome
32145358	Can natural language processing provide accurate, automated reporting of wound infection requiring reoperation after lumbar disc	Karhade AV, Bongers MER, Groot OC	2020	10.1016/j.spinee.2020.02.021	No	Outcome
35942941	Prediction models for carbapenem-resistant Enterobacterales carriage at liver transplantation: A multicenter retrospective study	Freire MP, Rinaldi M, Terrabuio DRB	2022	10.1111/tid.13920	No	Outcome
35726257	Machine-learning based prediction and analysis of prognostic risk factors in patients with candidemia and bacteraemia: a 5-year an	Gao Y, Tang M, Li Y, Niu X, Li J, Fu C,	2022	10.7717/peerj.13594	No	Outcome
32579600	Comprehensive integrated NGS-based surveillance and contact-network modeling unravels transmission dynamics of vancomycin-r	Neumann B, Bender JK, Maier BF, W	2020	10.1371/journal.pone.0235160	No	Outcome
37448774	Using multiple indicators to predict the risk of surgical site infection after ORIF of tibia fractures: a machine learning based study	Ying H, Guo BW, Wu HJ, Zhu RP, Liu	2023	10.3389/fcimb.2023.1206393	No	Outcome
35005696	Admissions to a Low-Resource Neonatal Unit in Malawi Using a Mobile App and Dashboard: A 1-Year Digital Perinatal Outcome Au	Mgusha Y, Nkhoma DB, Chiume M, C	2021	10.3389/fdgth.2021.761128	No	No new technologies
29576042	A Generalizable, Data-Driven Approach to Predict Daily Risk of Clostridium difficile Infection at Two Large Academic Health Centers	Oh J.; Makar M.; Fusco C.; McCaffrey	2018	10.1017/ice.2018.16	No	No new technologies
30922931	Development and validation of a semi-automated surveillance system-lowering the fruit for non-ventilator-associated hospital-acq	Wolfensberger A, Jakob W, Faes Hes	2019	10.1016/j.cmi.2019.03.019	No	No new technologies
34988091	Development and Internal Validation of Supervised Machine Learning Algorithms for Predicting the Risk of Surgical Site Infection Fo	Wang H, Fan T, Yang B, Lin Q, Li W, Y	2021	10.3389/fmed.2021.771608	No	Outcome
35057734	Spatiotemporal prediction of vancomycin-resistant Enterococcus colonisation	van Niekerk JM, Lokate M, Braakmar	2022	10.1186/s12879-022-07043-9	No	Outcome
35790097	Using Temporal Data Mining on Patient Data for Clinical Decision Making in the Care of the Sick Newborn	Tan S, Unnikrishnan KP.	2022	25	No	Outcome
35799456	A non-randomised pragmatic trial for the early detection and prevention of surgical wound complications using an advanced hydro	Sandy-Hodgetts K, Norman R, Edmor	2022	10.1111/iwj.13823	No	Outcome
33795708	Reorganization of nurse scheduling reduces the risk of healthcare associated infections	Valdano E, Poletto C, Boëlle PY, Coliz	2021	10.1038/s41598-021-86637-w	No	No new technologies
32308875	Using Natural Language Processing to improve EHR Structured Data-based Surgical Site Infection Surveillance	Shi J, Liu S, Pruitt LCC, Luppens CL, F	2020	21	No	Outcome
29893653	Validation of semiautomated surgical site infection surveillance using electronic screening algorithms in 38 surgery categories	Cho SY, Chung DR, Choi JR, Kim DM,	2018	10.1017/ice.2018.116	No	Outcome
37726843	Staphylococcus aureus surgical site infection rates in 5 European countries	Mellinghoff SC, Bruns C, Albertsmeie	2023	10.1186/s13756-023-01309-w	No	Outcome
150	An Application of Convolutional Neural Networks for the Early Detection of Late-onset Neonatal Sepsis	Hu, YF; Lee, VCS; Tan, K	2019	10.1109/IJCNN.2019.8851683	No	Outcome

32032262	Identification of Pediatric Sepsis for Epidemiologic Surveillance Using Electronic Clinical Data	Weiss SL, Balamuth F, Chilutti M, Ra	2020	10.1097/PCC.0000000000002170	No	No new technologies
33157307	Preventing infectious diseases in Intensive Care Unit by medical devices remote control: Lessons from COVID-19	Garzotto F, Comoretto RI, Osterman	2021	10.1016/j.jcrc.2020.10.014	No	Type of study
124	Modelling and Classification of Sepsis using Machine Learning	Amrita, I; Martis, RJ; Ashwini, K	2021	10.1109/ICEECCOT52851.2021.9707934	No	Outcome
34116215	External validation of a predictive model of adverse events following spine surgery	Fatemi P, Zhang Y, Han SS, Puringtor	2022	10.1016/j.spinee.2021.06.006	No	No new technologies
35047054	Prediction of Lung Infection during Palliative Chemotherapy of Lung Cancer Based on Artificial Neural Network	Guo W, Gao G, Dai J, Sun Q.	2022	10.1155/2022/4312117	No	Outcome
37163757	Comparison of Administrative versus Electronic Health Record-based Methods for Identifying Sepsis Hospitalizations	Karlic KJ, Clouse TL, Hogan CK, Garla	2023	10.1513/AnnalsATS.202302-105OC	No	Outcome
151	Big data-based grey forecast mathematical model to evaluate the effect of Escherichia coli infection on patients with lupus nephriti	Fan, MX; Gu, SS; Jin, YS; Ding, L; Gho	2021	10.1016/j.rinp.2021.104339	No	Outcome
36050996	Construct and Validate a Predictive Model for Surgical Site Infection after Posterior Lumbar Interbody Fusion Based on Machine Lea	Xiong C, Zhao R, Xu J, Liang H, Zhang	2022	10.1155/2022/2697841	No	Outcome
37773495	Use of Electronic Clinical Data to Track Incidence and Mortality for SARS-CoV-2-Associated Sepsis	Shappell, CN; Klompas, M; Chan, CS/	2023	10.1001/jamanetworkopen.2023.35728	No	Outcome
35962320	Harmonized procedure coding system for surgical procedures and analysis of surgical site infections (SSI) of five European countries	Mellinghoff SC, Bruns C, Al-Monajjec	2022	10.1186/s12874-022-01702-w	No	No new technologies
125	A Machine Learning-Based Missing Data Imputation with FHIR Interoperability Approach in Sepsis Prediction	Beltran, CFT; Ibañez, EDV; Orejuela,	2022	10.1007/978-3-031-23821-5_9	No	Outcome

Data Extraction Form

PMID	Title	Author	Journal	Year of publication	Study design	Country	Period of study	Duration (Months)	Last year of the study	Single/ Multicentre	Study aims/objectives	Setting of study	Other (specific) - ward	Digital technology classification	Concise description of digital technology (when/as described by authors)	Aim of technology	Intervention under surveillance	Infectious disease targeted by digital technology	Potential benefits to public health functions	Potential negative impacts to public health functions	Obstacles/barriers to implementation	Additional comments	Comparative study	Target population (N)	Sex (M)	Age	Comparative population	Sex (M)	Age	Pros and cons for the new technologies vs old one	Quality assessment tool	Quality value
29132696	Development and validation of an automated ventilator-associated event electronic surveillance system: A report of a successful implementation	Courtney Hebert Et Al.	American Journal of Infection Control	2018	Prospective observational	US	January 1, 2014 - June 30, 2014	6	2014	Single	-To describe the implementation of an electronic algorithm for ventilator associated events (VAEs) at a large academic medical center<br>-To describe the steps involved to develop an automated surveillance dashboard and discuss its effect on IP workflow	University	ICU	Health informatics	Using an automated algorithm, a VAE report is generated on each ventilated patient by automatically extracting the required data from discrete fields within the EHR, classifying all MV patients using the CDC/NHSN algorithm parameters for VAE surveillance, and populating a simple dashboard used by the IPs to perform VAP surveillance	Surveillance	Ventilator-associated pneumonias (VAPs)		Cases of VAP can be identified automatically and more accurately (lessened workload for IPs)<br>- The near–real-time identification of VAEs could potentially lead to interventions in high-risk patient care areas while The patient is still present on The unit to actually assess for possible process failures		The need to have an ongoing, close relationship among the IPs, information technology, the laboratory, and the data analyst, so as to allow ongoing updates and validation as EHRs, microbe nomenclature, and definitions evolve		Yes	Unknown			Same population			The VAE dashboard correctly classified all cases of possible and probable VAPs identified by manual surveillance, and identified additional cases of possible and probable VAPs that were missed by manual surveillance processes<br>- Workflow of the infection preventionists was simplified after implementation of the dashboard with subjective time savings reported	Adapted Quality Assessment Tool for Retrospective and prospective observational studies	Low Quality
29661634	Assessing patient risk of central line-associated bacteremia via machine learning	Cole Beeler Et Al.	American Journal of Infection Control	2018	Case-control	US	January 1, 2013 - May 31, 2016	41	2016	Multicentre	To describe the process of development and validation of a CLABSI (central line-associated bloodstream infections) risk prediction model at Indiana University Health Academic Health Center (IUH AHC)	University	All	Machine Learning	A machine learning model (random forest) that accurately predicts which current inpatients with a CVL are at risk (patient risk of CLABSI is stratified from highest to lowest), thus helping optimize daily clinical decision-making.	Surveillance	Central line-associated bloodstream infections (CLABSIs)		Machine learning can be used to develop accurate models to predict the risk of CLABSI in real time prior to the development of infection		- Alert fatigue could potentially result in poor sustainability<br>- The model is dependent on The accuracy of The EMR<br>- Efficacy of this model assumes an active IP workforce to review the scores and implement potential interventions		No	70218	33705	52 (mean)					Adapted Quality Assessment Tool for Retrospective and prospective observational studies	Low Quality
37100291	Using artificial intelligence to reduce orthopedic surgical site infection surveillance workload: Algorithm design, validation, and implementation in 4 Spanish hospitals	Álvaro Flores-Balado Et Al.	American Journal of Infection Control	2023	Retrospective observational	Spain	January 2014 - May 2021	89	2021	Multicentre	To present the design and validation of an algorithm for SSI detection after hip replacement surgery and to describe its successful implementation in 4 public hospitals in Madrid, Spain.	University	Orthopedy	Machine Learning	A multivariable algorithm, AI-HPRO, using natural language processing (NLP) and extreme gradient boosting to screen for SSI in patients undergoing hip replacement surgery to permit accurate, real-time orthopedic SSI surveillance	Surveillance							No	6741							Adapted Quality Assessment Tool for Retrospective and prospective observational studies	Low Quality
29854220	Electronic Surveillance For Catheter-Associated Urinary Tract Infection Using Natural Language Processing	Sanger et al.	AMIA Annual Symposium Proceeding Archive	2018	Prospective observational	US	February 2010 July 2011-august 2011 august 2012	29	2012	Single	To Improve the early identification of CAUTI	University	More than one	Natural Language processing	An automated surveillance tool capable of applying the NHSN definition as written, using NLP to capture subjective symptoms documented in free-text clinical notes. NPL algorithm working on a Microsoft Amalga UIS database	Surveillance	CAUTI		The early identification of CAUTI and so the reduction of time, infection-related deaths and cost (estimated time savings of <100 hours per year, estimated cost savings are up to $10,000 yearly)		This kind of approach is not feaseble on a large scale due to variability in documentation peactices and technical resources	sensitivity of 97.1%, specificy of 94.5%, PPV of 66.7% for identifying CAUTI.	No	1519	1019	56,1 (mean)				<u>PROS AND CONS</u>: Useful just as a screening method.	Adapted Quality Assessment Tool for Retrospective and prospective observational studies	Low Quality
29854116	Detecting Evidence of Intra-abdominal Surgical Site Infections from Radiology Reports Using Natural Language Processing	Chapman et al.	AMIA Annual Symposium Proceedings	2018	Retrospective observatio	US				Single	Develope an NLP system to automatically identify mentions of surgical site infections in radiology reports and classify reports containing evidence of surgical site infections leveraging these mentions	Other	Other	Natural Language processing	The overall approach for this study included four steps: (1) identifying surgical patients and collecting their associated EHR data; (2) annotating text data and developing a knowledge base; (3) detecting mentions of SSIs and classifying reports for the presence of a SSI; and (4) evaluating automated methods against the reference standard annotations for SSIs. Institutional review board approval (IRB) was obtained.	Surveillance	SSI		The automated detection of adverse surgical event has the potential to revolutionize how providers and hospitals detect and report quality measures. In addition, leveraging automated detection into clinical decision support services may lead to better surveillance and the potential to improve patient care.	There are several limitations to our study. We used reports from the MIMICIII Critical Care Database. These reports were written for surgical patients in intensive care. Because of the severity the patient's condition, the prevalence of surgical site infections is higher than what we would expect to see in the general population. Another limitation is the focused scope of this project. We looked only at radiology reports, specifically including only CT reports because of their relatively consistent structure and content. We focused on one specific anatomic region, and our targets included a relatively small lexicon of a single clinical concept, fluid collection, as evidence of surgical site infection. The CDC lists three additional criteria for defining an organ/space surgical site infection: purulent drainage from a drain, organisms found in a culture, and diagnosis of a surgical site infection by a surgeon or attending physician			No	505							Adapted Quality Assessment Tool for Retrospective and prospective observational studies	Low Quality
37350879	Developing an LSTM Model to Identify Surgical Site Infections using Electronic Healthcare Records	Amber C Kiser Et Al.	AMIA Summits on Translational Science Proceedings	2023	Retrospective observational	US	January 2016 - June 2021	66	2021	Single	To develope a deep learning model, an LSTM, to identify SSIs from the EHR	University	More than one	Machine Learning	A long short-term memory (LSTM) network is a variant of an RNN, a type of deep learning algorithm typically used in sequence prediction. LSTM models have the capability to produce predicted probabilities at each time step, an advantage when looking beyond classification to prediction in the future.	Surveillance	Postoperative surgical site infections		- Allowing automated surveillance of SSIs, which is a critical component of quality improvement mechanisms<br>- The implementation of machine learning models to assist in identification and extraction of data could cut overhead costs of both time and money		- The model only uses structured EHR data<br>- External validation is required		No	9185	4467						Adapted Quality Assessment Tool for Retrospective and prospective observational studies	High Quality
37143157	Validation of the first computerized indicator for orthopaedic surgical site infections in France: ISO-ORTHO	Grammatico-Guillon et al.	Antimicrobial Resistance & Infection Control	2023	Retrospective observational	France	Jan - 18<br>Sept - 18	9	2018	Multicentre	Validate an outcome indicator ISO_ORTHO by measuring its performance to detect SSI over the 90 days TH/TK	Other	Orthopedy	Health informatics	Automated indicator	Surveillance	SSI		Quality safety improvement		A limitation of indicators based on HDD is the lack of clinical and medical device data and the absence of a dynamic link between the occurrence of complications and electronic health records. In the absence of exhaustive clinical registries for THA and TKA, several things would be relevant to detect SSI, including patient follow up, automated complication monitoring from clinical, microbiological and imaging data warehouses.		No	143227							Adapted Quality Assessment Tool for Retrospective and prospective observational studies	Low Quality
37884948	The augmented value of using clinical notes in semi-automated surveillance of deep surgical site infections after colorectal surgery	Verberk et al.	Antimicrobial Resistance & Infection Control	2023	Retrospective observational	Sweden	Jan - 2015<br>Aug - 2020	68	2020	Single	To investigate the performance of this semi-automated surveillance algorithm augmented with a natural language processing (NLP) component to improve positive predictive value (PPV) and thus workload reduction (WR)	University	Other	Natural Language processing	A NLP algorithm was developed and validated. A list of keywords was compiled by reviewing clinical literature and local case reports, and by expert consultation in two EU States. From keywords list to lexicon list. Furthermore, a rule-based NLP component was developed based on keywords reflecting the deep SSI definition.	Surveillance	SSI		Improve positive predictive value<br>Reduction of the workload	Lack of trasparency	Developing and building NLP models require substantial effort of information technology experts		No	2675							Adapted Quality Assessment Tool for Retrospective and prospective observational studies	High Quality
37679824	External validation of semi-automated surveillance algorithms for deep surgical site infections after colorectal surgery in an independent country	van der Werff et al.	Antimicrobial Resistance &Infection Control	2023	Retrospective observatio	Stockholm /Sweden	1/01/2015-31/08/2020	68	2020	Single	Externally validate two existing semi-automated surveillance algorithms for deepSSI after colorectal surgery, developed on Spanish and Dutch data, in EHRdata of a Swedish academic hospital center	University	Other	Machine Learning	With semi-automated surveillance patients are dividedin low- and high-probability cases where low-probabilitycases are automatically regarded as no SSI whilehigh-probability cases undergo manual record review todetermine SSI status [6]. Two existing semi-automatedclassification algorithms to assess deep SSI and/or organ/space SSI, from here on together referred to as deep SSI,were validated. 1)Original classification algorithm of van Roodenet al., i.e., probability of having a deep SSI basedon (re)admission and discharge data, mortality, reoperations, radiology orders, antibioticprescriptions, and microbiology cultures. 2)Adapted classification algorithm according toVerberk et al., i.e., probability of having a deep SSIbased on the original classification algorithm withoutthe microbiology component	Surveillance	SSI						No	225	115	66 (median)					Adapted Quality Assessment Tool for Retrospective and prospective observational studies	High Quality
37658409	Development of machine learning models for the detection of surgical site infections following total hip and knee arthroplasty: a multicenter cohort study	Guosong Wu Et Al.	Antimicrobial Resistance and Infection Control	2023	Cohort	Canada	January 1st, 2013 - August 31st, 2020	92	2020	Multicentre	- To determine the incidence of SSI and to develop machine learning models to automate the process of detecting complex (deep incisional/ organ space) SSI following THA/TKA.	Tertiary non-university	Orthopedy	Machine Learning	XGBoost is a machine learning model that combines weak decision trees to perform regression and classification, using administrative data, EMR free text data and both types of data	Surveillance	Deep incisional SSIs, organ space SSIs and complex SSIs		- Applying these models has the potential to reduce the workload for chart reviews of traditional IPC surveillance programs<br>- The developed models could also be applied to clinical practice to support quality improvement initiatives locally, nationally, or internationally		- The success of the models largely depends on the availability and quality of data in each hospital's EMR system		No	22059	9543	66 (median)					Adapted Quality Assessment Tool for Retrospective and prospective observational studies	High Quality
34663246	Implementation of an automated cluster alert system into the routine work of infection control and hospital epidemiology: experiences from a tertiary care university hospital	Aghdassi et al.	BMC Infectious disease	2019	Retrospective observatio	Berlin, Ger	2019	12	2019	Single	Improve cluster detection and pathogen surveillance through the use of an automated cluster alert system	General Hospital	All	Health informatics	CLAR reviewed and analyzed routinely collected microbiological and patient-related data (e.g. patient movement) that converged and were stored in a data warehouse. CLAR utilized data of the previous two years from the data warehouse to calculate a baseline for every included pathogen at every hospital ward. Daily, this baseline was compared to data from a period of the previous 14days. If the baseline was exceeded, CLAR generated an alert email that was sent to the responsible IPC physician for review.	Surveillance		Acinetobacter baumannii, Clostridioides difficile, Citrobacter, E. Coli, Enterococcus, Enterobacter, Klebsiella, PA, SA, Salmonella, Serratia	The comprehensive approach of CLAR that includes susceptible organisms and monitors cluster occurrence in all wards of a hospital may show its true merit in situ- ations when human healthcare resources are scarce or need to be re-allocated. The COVID-19 pandemic has demonstrated how quickly human resources can become scarce and how demands on employees can change. To have a robust, autonomous system in place that ensures a continuous and steady quality of cluster moni- toring could be a great advantage in such situations.				No	Unknown							Adapted Quality Assessment Tool for Retrospective and prospective observational studies	Low Quality
36859254	ICU infection surveillance can be based on electronic routine data: results of a case study	Schaumburg et al.	BMC Infectious Disease	2023	Retrospective observational	GERMANY	April 2017 to March 2018	12	2018	Single	The objective of this study was to investigate how the results obtained using the EFFECT algorithm compare to those obtained via manual chart review and clinical documentation.	General Hospital	ICU	Health informatics	EFFECT algorithm	Surveillance	ICU-acquired primary bacteremia/sepsis ICU-acquired MDROs	***<u>MDRO</u>***: MRSA, VRE, MDRGN, ***<u>Bacteremia/Sepsis</u>***: common commensals, Staphylococcus aureus, Enterococcus faecium and Enterococcus faecalis	Hospital infection surveillance based on electronically generated routine data may be a worthwhile and sustainable alternative to manual chart review with less time and resources consuming			considerable concordance between manual documentation and the EFFECT algorithm. Approximately 75% of MDRO cases and 85% of cases of primary bacteremia/sepsis were classified as ICU-acquired by both manual chart review and EFFECT.	No	6460						***<u>Pros:</u>*** the flexibility of the algorithmic approach regarding different endpoint definitions and can be formatted to fit the needs of the observer.	Adapted Quality Assessment Tool for Retrospective and prospective observational studies	High Quality
34454414	Predicting postoperative surgical site infection with administrative data: a random forests algorithm	Yelena Petrosyan Et Al.	BMC Medical Research Methodology	2021	Case-control	Canada	April 1, 2010 - March 31, 2015	54	2015	Single	This study aimed to develop efficient algorithms to identify SSIs within 30 days after surgery, using Machine Learning algorithms on health administrative data	University	More than one	Machine Learning	Machine Learning approaches, including a Random Forests algorithm and the high-performance logistic regression, were used to derive parsimonious models to predict SSI status.	Surveillance	Postoperative surgical site infections		Machine learning approaches can integrate and utilize a large amount of administrative data so as to effectively identify SSIs		- External validation of this model is required before it can be routinely used to identify SSIs		No	14351	6266	56,7 (mean)					Adapted Quality Assessment Tool for Retrospective and prospective observational studies	High Quality
32029574	Validation of automated sepsis surveillance based on the Sepsis-3 clinical criteria against physician record review in a general hospital population: observational study using electronic health records data	Valik et al.	BMJ	2020	Retrospective observatio	Stoccolma	Lug-2012 dic-2013	6	2013	Multicentre	Develop and validate a fully automatec	General Hospital	All	Machine Learning	The primary objective of this study was to develop and validate a fully automated EHR based surveillance algorithm with respect to the review of medical records of physicians in non-intensive care units, using the clinical criteria Sepsis-3	Outbreak detection	Sepsi		it is possible to build a fully automated sepsis surveillance system based on Sepsis-3 clinical criteria, which correctly captures nearly 90 percent of sepsis episodes occurring outside the intensive care unit and assigns events to space (ward) and time (onset). The utility of the algorithm has been demonstrated by indicating variations in the incidence and mortality of hospital-derived sepsis by ward type, which can be used to inform infection prevention interventions and improve sepsis care.		Not all hospital admissions with suspected infection contained the measurements necessary to assess a complete SOFA score, resulting in missing data.		No	95858	47066	64 (median)				Pros: timeliness of detection<br>Cons: loss of unrecorded data	Adapted Quality Assessment Tool for Retrospective and prospective observational studies	High Quality
33367518	Outbreak of Pseudomonas aeruginosa Infections from a Contaminated Gastroscope Detected by Whole Genome Sequencing Surveillance	Sundermann et al.	Clinical Infectious Diseases	2021	Retrospective observational	US	Nov - 16<br>May - 19	31	2019	Single	Development and validation of the Enhanced Detection System for Healthcare-Associated Transmission (EDS-HAT), which utilizes machine learning of the electronic health record (EHR) to identify the transmission route responsible for outbreaks detected by WGS surveillance. To further investigate this approach, we retrospectively performed 2.5 years of WGS surveillance and combined these data with our ML algorithm. During this analysis, we uncovered a previously undetected Pseudomonas aeruginosa outbreak.	University	All	Machine Learning	Enhanced Detection System for Healthcare-Associated Transmission (EDS-HAT), which utilizes machine learning of the electronic health record (EHR) to identify the transmission route responsible for outbreaks detected by WGS surveillance.	Outbreak detection		Pseudomonas	Enhancing outbreak detenction		Inconsistencies in charge codes		No	Unclear							Adapted Quality Assessment Tool for Retrospective and prospective observational studies	Low Quality
34791136	Whole-Genome Sequencing Surveillance and Machine Learning of the Electronic Health Record for Enhanced Healthcare Outbreak Detection	Sundermann et al.	Clinical Infectious Diseases	2022	Retrospective observatio	US	Nov - 2016<br>Nov - 2018	25	2018	Single	To performed WGS surveillance of healthcare-associated bacterial pathogens, using EHR ML to identify the transmission routes for WGS-detected outbreaks, which were investigated by an IP expert. Potential infections prevented were estimated and compared with traditional IP practice.	University	All	Machine Learning	The ML algorithm scores each outbreak by the maximum log-likelihood ratio of observing the case infections, given that exposure to the principal transmission route probabilistically causes infection over the likelihood of a nontransmission explanation	Surveillance		Acinetobacter species, Pseudomonas species, extended-spectrum B-lactamase-producing (ESBL) Escherichia coli, Klebsiella species, Clostridioides difficile, ESBL Enterobacter species, vancomycin-resistant Enterococcus (VRE), methicillin-resistant Staphylococcus aureus (MRSA), Stenotrophomonas species, Serratia species, Burkholderia species, Legionella species, Providencia species, Proteus species, and Citrobacter species.	Higher sensitivity<br>cost saving				Yes	297			133				Adapted Quality Assessment Tool for Retrospective and prospective observational studies	High Quality
32745591	A passive monitoring tool using hospital administrative data enables earlier specific detection of healthcare-acquired infections	Rewley et al.	Elsevier	2020	Retrospective observatio	UK	1/1/2011 al 1/1/2015	48	2015	Multicentre	whether the amount of time a patient spends on the ward with other patients clinically suspected of infection, termed "co-presence," can be used as a tool to predict subsequent healthcare-associated infection	General Hospital	All	Health informatics		Surveillance		MRSA, E.Coli, PA, C.diff., Norovirus	This study demonstrated that the number of co-presence hours with patients clinically suspected of being infected is a measure that serves as a screening test of infection. In addition, it showed that the use of co-presence time as a screening test can allow detection of infection resulting from interpersonal transmission in the healthcare setting up to 22 hours earlier.		co-presence makes no assumptions about how the pathogen is passed from one person to another, whether directly, carried by a third person or deposited on an object and picked up by another person. This prevents the prediction of nosocomial infections that do not result from interpersonal transmission.		No	133304	59987	56 (mean)					Adapted Quality Assessment Tool for Retrospective and prospective observational studies	High Quality
36470694	Development and validation of models for detection of postoperative infections using structured electronic health records data and machine learning	Colborn et al.	Elsevier	2023	Retrospective observational	US	30/06/2013-1/03/2019	69	2019	Multicentre	To create an automated,reliable, and generalizable method to identify postoperative infections,including SSI, UTI, pneumonia, and sepsis/septic shock,using machine learning applied to EHR data.	University	Other	Machine Learning	Structured electronic health records data were linked to postoperative outcomes data from theAmerican College of Surgeons National Surgical Quality Improvement Program. Predictors from theelectronic health records included diagnoses, procedures, and medications. Infectious complicationsincluded surgical site infection, urinary tract infection, sepsis, and pneumonia within 30 days of surgery.The knockoff filter, a penalized regression technique that controls type I error, was applied for variableselection. Models were validated in a chronological held-out dataset.	Surveillance	Pstoperative infections (SSI,UTI, PNEUMONIA, SPESIS/septic shock)		artificial intelligence can be used for the surveillanceof postoperative infections with high accuracy, specificity,and sensitivity using EHR data and simple linear regression coefficients.Although the model selection technique that was appliedwas complex, the final chosen models are simple to implement.Unlike HAI surveillance that relies on manual chart review, thesemodels can be applied to all operations and results can bedisseminated in a timely manner.		Due to the low PPV, these modelsare most appropriate for tracking rates of complications over time,but they may require additional manual review to identify exactpatients who experienced an infection.		No	30639	13450	55.3 (mean)					The Newcastle-Ottawa Scale	High Quality
33742085	Remote assessment of surgical site infection (SSI) using patient-taken wound images: Development and evaluation of a method for research and routine practice	Macefield et al	Elsevier (Journal of Tissue Viability)	2023	Prospective observational	UK	January 2019- June 2019	6	2019	Single	The aim of this study was to i) develop instructions for patients or family members/caregivers to take a clear,standardised image of the surgical wound after hospital discharge and aprocess for transmitting them, and ii) evaluate the acceptability andusability of the method and quality of the images for remote SSIassessment.	General Hospital	Other	Smartphone and tablet computing devices	This study was conducted in two phases, drawing on methodologicalapproaches for developing and validating patient-reported outcomemeasures and evaluating human-system interaction [32–34]. Phase Iinvolved a literature review to identify the content for wound photographyinstructions for patients, followed by cognitive interviews andobservations to pre-test and refine the instructions and the process totransmit images. Phase II was a prospective cohort study to evaluate themethod remotely and assess image quality. A personalised hyperlink to access the online survey was sent by email tocoincide with receiving the photography instructions by post. Includedin the survey were step-by-step instructions on how to upload andtransmit the wound image (or multiple images if patients had more thanone wound), items to collect data on patients' familiarity and experiencewith technology and a set of 'debriefing' questions to collect informationon how long it took to take the wound images and whether help wasrequired. A box for free-text responses was also provided. Reminderemails or telephone calls were made to those who had not respondedwithin five days.A sub-sample of participants were selected for a follow-up telephonecall to elicit further information on any problems encountered whentaking and transmitting images remotely.	Surveillance	SSI		Digital images are moving towards being part of standard care with increasing recognition of the benefits for health care providers and patients. With aneffective system in place to collect and analyse data from such anoutcome measure in real time, such as automated notification of scoresover a pre-defined threshold, remote assessment has the potential toidentify problems sooner before they extenuate. This may lead to fastertreatment compared to the delay that may occur when schedulingin-person appointments.		Most patients were white/white British, and the acceptability and feasibility of the method in other ethnic or cultural groups is unclear		No	89	49						Cochrane Risk of Bias (RoB 2)	High risk of Bias
35372195	A Data-Driven Framework for Identifying Intensive Care Unit Admissions Colonized With Multidrug-Resistant Organisms	Çağlayan et al.	Frontiers in Public Health	2022	Retrospective observational	US	2017<br>2018	24	2018	Single	Develop a modeling framework that can cope with significant class imbalance, commonly observed in clinical datasets, and can be used (1) to generate timely and accurate predictions for newly admitted ICU patients, and (2) to identify the key sociodemographic and clinical factors affecting the incidence of MDRO colonization.	University	ICU	Machine Learning	Accurate and interpretable framework for predicting VRE, CRE, and MRSA colonization upon ICU admission. The developed predictive analytics framework achieved the following sensitivity and specificity values for VRE, CRE, and MRSA colonization: 80% and 66% for VRE with LR, 73% and 77% for CRE with XGBoost, and 76% and 59% for MRSA with RF. Further, we predicted MDRO (i.e., VRE, CRE, or MRSA) colonization as an aggregate outcome with 82% sensitivity and 83% specificity for MDRO using RF.	Surveillance		VRE<br>CRE<br>MRSA	Selective and timely use of infection control measures in ICUs.<br>Positive impact on LTCF.		Isolation of the informative system (no linkage to extra-hospital informations).<br><br>Prediction models have been previously reported to perform worse when they are implemented in clinical practice and applied to new individuals that are different than the original study population that the model was derived		No	3958							Cochrane Risk of Bias (RoB 2)	Low risk of Bias

37581167	Using Machine Learning to Predict Surgical Site Infection After Lumbar Spine Surgery	Chen et al.	Infection and Drug Resistance	2023	Retrospective observational	CHINA	June 2012 to February 2021	102	2021	Single	To select the ideal clinical variables using various machine learning algorithms and their intersection to build an ideal prediction model and perform internal verification. This prediction model might guide clinical diagnosis and prevention.	General Hospital	Other	Machine Learning	Data were randomized into the test and verification groups. In the test group, specific variables were screened via logistic regression analysis, Lasso regression analysis, support vector machine (SVM), and random forest. Specific variables acquired using these four methods were intersected, and a dynamic model was constructed. ROC and calibration curves were constructed to assess model performance. Finally, using the verification group, model performance was verified internally using ROC and calibration curves. Three machine learning models were employed to filter variables, and their validity was assessed using the verification group.	Surveillance	SSI		To anticipate the potential occurrence of SSIs in patients prior to surgery. In cases where a high risk is identified, appropriate intervention measures can be implemented before surgery.The goal is to mitigate the risk of postoperative SSIs, facilitate patients' speedy recovery, and alleviate unnecessary financial burdens	No	The machine learning algorithm model is based on data from a single center; as a result, this model might not be applicable to other centers		No	1327	604	61 (mean)					Adapted Quality Assessment Tool for Retrospective and prospective observational studies	High Quality
36111457	Extending outbreak investigation with machine learning and graph theory: Benefits of new tools with application to a nosocomial outbreak of a multidrug-resistant organism	Atkinson et al.	Infection Control & Hospital Epidemiology	2023	Retrospective observational	Switzerland	Jan-18 Jul-20	31	2020	Single	• What are the risk factors for VRE colonization? • Which patients should be screened? • What are the "hot spots"in terms of devices, rooms and employees where transmission may have occurred? • What is the potential benefit of a contact screening approach based on the network graph approach, compared to the traditional proximity-based contact screening?	Tertiary non-university	All	Machine Learning	A machine-learning "decision tree" approach was used to identify a VRE positive individual. In decision-tree learning, a tree-like model of decisions and subsequent outcomes is chosen, which models the path from observations to conclusions. A network graph model involving the identified risk factors was developed. We identified potential transmission hotspots: healthcare employees with frequent VRE patient contact, rooms in which VRE patients were present, and medical devices used in conjunction with diagnosing and treating these patients. The network graph analysis used eigenvector centrality to generate a daily "hotspot list", which was sent to the relevant organizational unit involved in disinfecting rooms and medical devices.	Outbreak detection		VRE	Reduce un-necessary screening Tool for data-driven outbreak monitoring		Lacking of a hospitalwide, fully integrated, data warehouse		Yes	560	346	73 [63-82]	86684	44019	58 [33-75]	No	Adapted Quality Assessment Tool for Retrospective and prospective observational studies	Low Quality
31445292	A mobile app for postoperative wound care after arthroplasty: Ease of use and perceived usefulness	Scheper et al.	International Journal of Medical Informatics	2019	Prospective observational	NETHERLANDS	July to December 2017	6	2017 / 2018 (not declared by authors)	Multicentre	Primary objective was toinvestigate the ease of use and perceived usefulness of using a woundcare app. Secondary objectives were the number of alerts created, the amount of days the app was actually used and patient-reported wound infection.	Other	Orthopedy	Digital health/e-health/m-health	A mobile woundcare app with an integrated algorithm to detect complications may lead to improved monitoring and earlier treatment of complications.	Surveillance	prosthetic joint infection (PJI)		The app may be cost-effective by preventing prosthetic joint infection (PJI). . The costs of revision surgery forone patient (estimated costs around 30.000 euro) are about the same as the costs for the development of this app.			Good compliance is needed in order to really benefit from the app. Of all included patients, 59% used the app as intended until day 30. One of the main understandable -reasons for discontinuation was that patients deemed further use of the App irrelevant,since their postoperative recovery went uneventful. For these patients, further use of the app would obviously not have resulted in improved clinical outcome.	No	69	23	68 (median)					Adapted Quality Assessment Tool for Retrospective and prospective observational studies	High Quality
32955571	Novel Method to Flag Cardiac Implantable Device Infections by Integrating Text Mining With Structured Data in the Veterans Health Administration's Electronic Medical Record	Mull et al.	JAMA Network Open	2020	Cohort	Us	1/10/2016-31/09/2017	12	2017	Multicentre	To develop a method that includes text mining of electronic clinical notes to reliably and efficiently measure HAIs for CIED procedures	Other	Other	Machine Learning	Using electronicmedical record data for patients undergoing CIED procedures in Veterans Health Administration (VA) an algorithm to flag cases with a true CIED-relatedinfection based on structured (eg, microbiology orders, vital signs) and free text diagnostic andtherapeutic data (eg, procedure notes, discharge summaries, microbiology results) was developedand validated. Procedure data were divided into development and validation data sets. Criterionvalidity (ie, positive predictive validity [PPV], sensitivity, and specificity) was assessed via criterionstandardmanual medical record review.	Surveillance	SSI		The findings of this study suggest that the method of combiningstructured and text data in VA electronic medical records can be used to expand infectionsurveillance beyond traditional boundaries to include outpatient and procedural areas	Our study has several limitations.We conducted this study in the closed VA health care system,where the vast majority of patients (>97%) return for postoperative and postprocedural care. these findings mayoverestimate the predictive value of the algorithm in nonclosed health care settings, in whichpatients may receive subsequent care away from the initial health care system. This may be aparticularly large limitation in rural settings, where patients may need to travel long distances to atertiary care facility that performs CIED procedures but then return to a local facility with fewerservices for subsequent care.			No	19212	18766	71.8 (mean)					Adapted Quality Assessment Tool for Retrospective and prospective observational studies	Low Quality
32538798	Artificial Intelligence-Based Multimodal Risk Assessment Model for Surgical Site Infection (AMRAMS): Development and Validation Study	Chen et al.	JMIR Medical Informatics	2020	Retrospective observational	China	Jan - 2014 Jun - 2019	54	2019	Single	To develop an AI-based Risk Assessment Model for SSI based on routinely collected clinical data from an EMR system and modern machine learning techniques.	University	All	Machine Learning	Reprocessing of routinely collected data. Using the fastText algorithm, a first word embeddings based on a large Chinese corpus for further text-information encoding was generated. The conventional machine learning methods analyzed in this study included LASSO (least absolute shrinkage and selection operator) logistic regression with L1 penalty, random forest, and gradient boosting decision tree (GBDT), implemented by the XGBoost framework	Surveillance	SSI		Personalized guidance for the preoperative intervention of SSI.		Te model doesn't consider SSI diagnosed after discharge.	Digital technology classification: Machine learning + AI	No	21611	8318	54.3 (IQR 44-65)				SSI RAMs based on clinical data from an EMR system and modern machine learning techniques could identify high-risk patients more accurately than the old-fashioned NNIS risk index.	Adapted Quality Assessment Tool for Retrospective and prospective observational studies	High Quality
33548370	The accuracy of fully automated algorithms for surveillance of healthcare-associated urinary tract infections in hospitalized patients	S D van der Werff Et Al.	Journal of Hospital Infection	2021	Retrospective observational	Sweden	2010-2013	36	2013	Single	To develop a fully automated rule-based surveillance algorithm using EHR data for the detection of HA-UTI in hospitalized patients, and validate it against manual record review	University	All	Natural Language processing	A fully automated surveillance algorithm for HA-UTI e based on positive urine culture combined with simple NLP for UTI symptom detection and negation of fever when being the sole symptom, and using EHR data	Surveillance	Healthcare-associated urinary tract infections		- Such an algorithm may be used for surveillance purposes and could be implemented to replace manual surveillance by record review - the NLP method used is one of the simplest methods and should be less troublesome to apply in different settings than more complicated methods		- the algorithms were developed in a copy of EHR data, they have not yet been implemented and evaluation in real-life settings is warranted. Additionally, the algorithms need to be tested in other hospitals/EHR systems to assess generalizability - this algorithm is not directly applicable to other languages		No	15986							The Newcastle-Ottawa Scale	Fair Quality
36642336	Image-based digital post-discharge surveillance in England: measuring patient enrolment, engagement, clinician response times, surgical site infection, and carbon footprint	Rochon et al.	Journal of Hospital Infection	2023	Prospective observational	UK	giu-22	1	2022	Multicentre	To determine success in patient enrolment and engagement including reasons for non-response, the time for clinicians to respond to patients, SSI rates, and carbon emissions when conducting PDS using patient smartphones.	Tertiary non-university	Other	Smartphone and tablet computing devices	Patients receive a secure SMS text at pre-programmed intervals asking them to upload images (photographs) of their wound and provide information on wound healing. Patients can also upload wound images/information at any time if they have a concern.	Surveillance	SSI		Efficient management of SSI Patients' empowerment Reduction of carbon footprint of SSIs	Misinterpretation of results	Patients not owning a smartphone. Language difficulties		Yes	138	113	62.8 (23-84)	20	17	66.9 (51-83)	No	Adapted Quality Assessment Tool for Retrospective and prospective observational studies	High Quality
36913981	Clusters of healthcare-associated SARS-CoV-2 infections in Norwegian hospitals detected by a fully automatic register-based surveillance system	Skagseth et al.	Journal of Hospital Infection	2023	Retrospective observational	Norway	Mar - 20 Ago - 22	30	2022	Multicentre	Develop a register-based system to identify clusters of healthcare-associated COVID-19 in Norwegian hospitals	Other	All	Health informatics	During the pandemic, the NIPH established an emergency preparedness register e Beredt C19 e to collect and produce timely information on the pandemic. The institute has previously shown that by implementing different registerbased algorithms, it is possible to detect HAI of SARS-CoV-2 in hospitals and outbreaks of SARS-CoV-2 in long-term care facilities. Beredt C19 contains several different national registers, which epidemiologists at the NIPH can access and extract pseudonymized data from. Each team can only access data relevant to their analyses.	Outbreak detection		COVID-19	Identification of intra-hospital outbreaks in near real-time Automation of previously manual tasks for infection control specialist	Reduction of specificity, possibly leading to unnecessary attention from infection prevention and control specialists. One of the disadvantages of this system is that it is hard to verify whether the results are actual outbreaks or sporadic cases among patients and newly discharged patients discovered through screening	Infections in staff were not included in this study as we were not able to link staff to specific wards using the data available to us. From the moment that one of the biggest problems in national surveillance is the heterogeneity of the data structure for different institutions and regions, implementation in countries that, on the contrary of Norway, don't have a publicly owned central health registrers wich make data structure homogeneous		Yes	5033	2722	70 (median)	Unknown				Adapted Quality Assessment Tool for Retrospective and prospective observational studies	High Quality
35868812	Prediction of hospital-onset COVID 19 infections using dynamic networks of patient contact: an international retrospective cohort study	Myall et al.	Lancet Digit Health	2022	Cohort	UK, Switzerland	April 1, 2020, and April 1, 2021	12	2021	Multicentre	Predicting which patients are at risk of health-care-associated infection (HCAI)	General Hospital	More than one	Machine Learning	We used network analysis in combination with machine learning to predict patient-level HOCI using routinely captured hospital data.	Outbreak detection		COVID 19	Patients at low risk of infection acquisition could be potentially moved back to regular patient management faster, saving resources that are in demand. It can be applied to respiratory virus transmission in hospital, using widely available patient bed records		Staff and visitor contact data are not usable due to privacy restrictions.		No	51.157						*PROS:* HOCIs could be identified at the earliest opportunity.that dynamic networks of patient contacts can aid personalised predictions of infection. The predicted risk score for each patient can be used by clinicians to rank and prioritise *CONS:* the used contact definitions might not fully capture transmission	Adapted Quality Assessment Tool for Retrospective and prospective observational studies	High Quality
30877683	Deep Learning versus Conventional Machine Learning for Detection of Healthcare-Associated Infections in French Clinical Narratives	Sara Rabhi Et Al.	Methods of Information in Medicine	2019	Retrospective observational	France	October 2009 - December 2010	15	2010	Multicentre	To compare the performances of health care-associated infection (HAI) detection between deep learning and conventional machine learning (ML) methods using textual electronic medical records in French medical reports	University	More than one	Machine Learning	A convolutional neural network (CNN) is a type of artificial neural network. This study demonstrated the feasibility to use CNN as an automated classification model for HAI detection.	Surveillance	HAI		Automated HAI detection.				No	1531							Adapted Quality Assessment Tool for Retrospective and prospective observational studies	High Quality
31370028	Machine learning applications for the prediction of surgical site infection in neurological operations	Tunthanathip et al.	Neurosurgical Focus	2019	Retrospective observational	Thailand	2010 - 2017	96	2017	Single	Compare the performance of various ML models in terms of predicting surgical infection after neurosurgical operations	University	Other	Machine Learning	Training and testing a ML alghorithm to predict surgical infections	Surveillance	SSI		Predicting SSI		Not excellence performance	Comparation amond several ML models	No	1471	839						Adapted Quality Assessment Tool for Retrospective and prospective observational studies	Low Quality
34892341	Use of Convolutional Neural Nets and Transfer Learning for Prediction of Surgical Site Infection from Color Images	Fletcher et al.	No Journal / it is a paper for a conference 2021 43rd Annual International Conference of the IEEE Engineering in Medicine & Biology Society (EMBC)	2021	Quasi-experimental	Rwanda	Mar - 2017 Oct - 2017	8	2017	Single	To develop two deep learning models to predict wound infection, particularly addressing the problems of class imbalance and overfitting	University	Maternity ward	Machine Learning	Convolutional Neural Nets designed to detect an infection in a surgical wound using a color image taken from a mobile device.	Surveillance	SSI		Manage SSI in low resources areas	low sensitivity and class imbalance	Ambient lighting skin of the patient (colour)		No	729	0						Adapted Quality Assessment Tool for Retrospective and prospective observational studies	High Quality
34795398	Remote diagnosis of surgical-site infection using a mobile digital intervention: a randomised controlled trial in emergency surgery patients	McLean et al.	NPJ Digital Medicine	2021	RCT	UK	July 2016 to March 2020	45	2020	Multicentre	The objective of this study is to investigate whether a smartphone-delivered wound assessment tool results in earlier diagnosis and treatment of SSI after emergency abdominal surgery. Secondary aims include the evaluation of the impact of this intervention on healthcare services and patient experience of postoperative care.	Tertiary non-university	Other	Digital health/e-health/m-health	A smartphone-delivered wound assessment tool for earlier diagnosis and treatment of SSI after emergency abdominal surgery	Surveillance	SSI		The potential to improve access and perceived quality of care.			Disadvantaged groups who potentially stand to benefit most from improved access to care from telehealth (such as those elderly,status who are or from low socioeconomic or minority ethnic backgrounds) may lack sufficient digital access and/or literacy to gain equitably from these interventions.	Yes	223	106	41,8 (mean)	269	120	46,7 (mean)	The smartphone group was superior to routine care for patients in improving access and perceived quality of care post-discharge, while reducing the rate of community care attendance. Disadvantaged groups who potentially stand to benefit most from improved access to care from telehealth (such as those elderly,status who are or from low socioeconomic or minority ethnic backgrounds) may lack sufficient digital access and/or literacy to gain equitably from these interventions.	Adapted Quality Assessment Tool for Retrospective and prospective observational studies	High Quality
37147462	Evaluation of remote digital postoperative wound monitoring in routine surgical practice	McLean at al.	NPJ Digital Medicine	2023	Cohort	UK	1st July 2021 and 30th April 2022	10	2022	Multicentre	This study aimed to pilot a remote digital postoperative wound monitoring service and evaluate the readiness for implementation in routine clinical practice.	General Hospital	Other	Smartphone and tablet computing devices	Enrolled patients had a personal hyperlink to the online form automatically sent by short-messaging system (SMS) to their smartphones. Patients could complete the online form (tool) throughout the early postoperative period (postoperative day 1–30), This included an image of their surgical wound(s), and a series of patient-reported outcomes (PROMs) related to signs and symptoms of SSI	Surveillance	SSI		The chance of moving towards scheduled postoperative follow-up for only high-risk patients, limiting opportunities for healthcare interaction if not actively sought	the lack of direct, two-way patient-clinician communication.	Patients not owning a smartphone. No internet access. Language difficulties	This validates the approach adopted by the intervention here: a triage tool to enhance existing healthcare pathways, rather than to replace in-person assessment.	No	200	97	48				*PROS:* no issues reported with feasibility of the technology, with the reliability and quality of the interface rated highly. Patient acceptance was similarly high with regards to ease of use, satisfaction and usefulness. *CONS:* the desire for more frequent and personalised interactions. the lack of direct, two-way patient-clinician communication.	Adapted Quality Assessment Tool for Retrospective and prospective observational studies	High Quality
31978086	Lean back and wait for the alarm? Testing an automated alarm system for nosocomial outbreaks to provide support for infection control professionals	Schröder et al.	Plos One	2020	Prospective observational	Berlin, Germany	1/1/2016 - 31/12/2017	12	2017	Single	Evaluate a newly developed automatic outbreak detection system (AODS)	General Hospital	All	Machine Learning	Detect infection folocai by analyzing microbiological tests using three categories of algorithms: prediction intervals, statistical process control, and statistical modeling.	Outbreak detection		All	Use of AODS results in immediate alarm and allows earlier detection of an outbreak than manual detection		AODS can detect only possible clonal outbreaks. There is no gold standard for outbreak detection because there is no international consensus on the definition of outbreak. Therefore, specificity could not be calculated for our approach. Another limitation concerns clonal typing, which was not routinely performed.		No	Unknown						Pros: timeliness of outbreak detection Cons: not specific	Adapted Quality Assessment Tool for Retrospective and prospective observational studies	High Quality
37082820	Machine Learning Model for Assessment of Risk Factors and Postoperative Day for Superficial vs Deep/Organ-Space Surgical Site Infections	Wardah Rafaqat Et al.	Sage	2023	Retrospective observational	Pakistan	January 2019 - Dicember 2020	24	2020	Single	Machine learning (ML) algorithms offer the opportunity to analyze multiple factors to predict the type (surface or deep and organ space) and timing of SSI development.	University	Other	Machine Learning	The NSQIP database is a validated, institution-based surgical registry of patients' risk factors and 30-day postoperative outcomes. Six ML algorithms were trained as predictors of infection type (superficial vs deep/organ space) and time to infection, and tested using the area under the receiver operating characteristic curve (AUC-ROC).	Surveillance	Postoperative surgical site infections		Deep and organ-space infections have a greater impact on patient outcome than superficial SSIs. This study validated an assessment method differentiates superficial SSIs from deep or organ-space SSIs so that efforts to prevent deep or organ-space SSIs can be rationalized, especially in resource-limited settings.		A major limitation of this study was the limited sample size of patients included. In addition, because of the retrospective design of the study, data were not available for some variables and significant preprocessing of data was necessary		No	113	65	52,2 (mean)					The Newcastle-Ottawa Scale	High Quality
32210289	Development and Validation of a Semi-Automated Surveillance Algorithm for Cardiac Device Infections: Insights from the VA CART program	Archana Asundi et al.	Scientific Reports natureresearch	2020	Retrospective observatio	US	1/01/2008-31/12/2015	95	2015	Multicentre	Develop and validate a surveillance tool for CIED infection surveillance using structured data elements in order to detect infections and triage cases for manual review	Other	Other	Machine Learning	This study used structured data elements along the diagnostic and treatment pathway ofCIED infection management from the VA EHR captured in the CDW to develop an algorithm to detect infections.We used a limited definition of structured data focusing on diagnostic testing orders, laboratory results,pharmacy and billing data that is structured and standardized across all EHRs in order to ensure broad potentialapplicability of the detection algorithm. A sample of cardiac procedures collected as part of the VA ClinicalAssessment Reporting and Tracking (CART) quality program underwent manual review for the presence ofinfection; these cases were used in the development and validation of the semi-automated tool.	Surveillance	SSI		The electronic surveillance tools based onstructured data elements can be developed with high sensitivity and specificity and have the potential to expandmeasurement with audit and feedback to clinical areas that have not been served by traditional surveillance programs. Future improvements could include strategies to enhance detection of variables primarily captured inclinical notes.	Healthcare systems and practices are not static, but rather constantly evolving. Thus, automated infectionsurveillance algorithms that leverage clinical pathways, including this CIED infection flagging tool, will require ongoing adaption and updates as novel therapeutics are introduced and diagnostic strategies evolve. Thus,detection algorithms must move toward a learning health system model, with constant modifications and adaptationsto maintain their estimative utility			No	5753	2024						Adapted Quality Assessment Tool for Retrospective and prospective observational studies	High Quality
36350558	Can we automate spine fusion surgical site infection data capture?	Bauer et al.	Spine Deformity	2023	Retrospective observational	US	2012 - 2018	84	2018	Single	Create an electronic medical record (EMR) algorithm to automatically capture spine fusion SSI and compared its accuracy against the administrative chart review and self-reported morbidity and mortality (M&M) rates.	Tertiary non-university	Other	Electronic health records (EHRs)	Definition of an EMR algorithm to find SSI in spine fusion	Surveillance	SSI		High sensitivity detection of SSIs in pediatric population with spine fusion	Reduction of institutional rankings, prestige of structure and doctors.		orthopedic primary care infectious disease ER ipatient visits	Yes	914			914			Pro: high sensitivity for a potentially severe infection Cons: higher rate of false positive could impact on prestige and institutional ranking.	Adapted Quality Assessment Tool for Retrospective and prospective observational studies	High Quality
32105569	Developing a Surgical Site Infection Surveillance System Based on Hospital Unstructured Clinical Notes and Text Mining	Ciofi Degli Atti et al.	Surgical Infection (Larchmt)	2020	Retrospective observational	ITALY	First quarter of 2016 to the fourth quarter of 2018	36	2018	Single	The aim of the present study was to assess the accuracy of a text-searching algorithm to detect SSIs in children based on the application of regular expressions of unstructured clinical notes collected through different information systems compared to the standard active surveillance.	General Hospital	Other	Health informatics	A customized application to analyze clinical notes and code descriptions applying a pattern-matching algorithm based on regular expressions. The application was implemented adopting the Qt It also provides an embedded regular expression engine that enabled us to implement the algorithm presented easily to capture an SSI within the explored narrative texts. The algorithm was based on a set of search items that suggest the presence of an infection within 30 days after surgery.	Surveillance	SSI		This semiautomated surveillance has the potential of drastically reducing the workload of conventional surveillance, which implied active contact with all families		Electronic medical records is required	Compared with the total number of SSIs used as a reference standard, both methods had a specificity of 100%, a positive predictive value of 100%, and a negative predictive value >99.5%. Sensitivity was 70% for the text-mining algorithm and 60% for the active surveillance. Accuracy was >99% with both methods.	Yes	3.434			Same population			*PROS:* the completeness of the patients included in the denominator. In fact, the active follow-up surveillance based on telephone interviews of parents failed to trace 14% of the families *CONS:* The text-searching algorithm explored only the electronic components of the medical records.Validation of the tool also should be conducted.	Adapted Quality Assessment Tool for Retrospective and prospective observational studies	Low Quality
31186183	Monitoring Surgical Incision Sites in Orthopedic Patients Using an Online Physician-Patient Messaging Platform	Zhang et al.	The Journal of Arthroplasty	2019	Retrospective observational	US	January 2015 - June 2017	30	2017	Single	To determine the utility of wound pictures in identifying and managing wound abnormalities after hip and knee arthroplasty. We hypothesized that (1) younger patients would be more likely to use the mHealth platform, (2) patients and providers would be willing to make decisions regarding the necessity of in-person evaluation based on photographic findings, (3) normal photographic findings would reliably predict normal findings at subsequent in-person examination, and (4) remote digital wound evaluation could avoid an in-person evaluation of a normal appearing wound or prompt emergent evaluation of a wound with a concerning appearance.	General Hospital	Orthopedy	Digital health/e-health/m-health	This web-based and mobile platform was introduced to facilitate patient education and communication during the preoperative and postoperative period. Patients were provided with electronic care instructions, video-based rehabilitation programs, and real-time alerts to complete surveys assessing pain, function, and overall health; survey results and progress were viewable by their providers. The software was used for secure electronic text and image messaging between patients and providers, allowing patients to send wound images directly from their smartphones.	Surveillance	SSI		The sharing of abnormal wound images online between patients and their providers can prompt rapid evaluation and management of wound issues. In the case of normal appearing wounds, remote reassurance can help patients avoid unnecessary clinic and hospital visits for wound checks		Patient engagement, especially with older patients.		No	1467	544	65.2 (mean)					Adapted Quality Assessment Tool for Retrospective and prospective observational studies	High Quality
31596819	Natural Language Processing for the Identification of Surgical Site Infections in Orthopaedics	Thirukumaran et al.	The Journal of bone & Joint Surgery	2019	Retrospective observatio	US	1/01/2011-31/06/2017	78	2017	Single	To determine whether automating the abstraction process with natural language processing(NLP)-based models that analyze the free-text notes of the medical record can identify surgical site infections withpredictive abilities that match the manual abstraction process and that surpass surgical site infection identification from administrative data.	University	Orthopedy	Natural Language processing	We used surgical site infection surveillance data compiled by the infection prevention team to identify surgicalsite infections among patients undergoing orthopaedic surgical procedures. Wecompiled a list of keywords suggestive of surgical site infections, and we usedNLP to identify occurrencesof these keywords and their grammatical variants in the free-text notes of the medical record. The key outcome was a binaryindicator of whether a surgical site infection occurred. We estimated 7 incremental multivariable logistic regression modelsusing a combination of administrative and NLP-derived variables. We split the analytic cohort into training (80%) and testingdata sets (20%), and we used a tenfold cross-validation approach.	Surveillance	SSI		This study examines NLP's potential to automate the identification of surgical site infections. Thisautomation can potentially aid the prevention and early identification of these surgical complications, thereby reducingtheir adverse clinical and economic impact				No	1579	819	45.8 (mean)					Adapted Quality Assessment Tool for Retrospective and prospective observational studies	High Quality
33676936	A machine learning approach to predict healthcare-associated infections at intensive care unit admission: findings from the SPIN-UTI project	Barchitta et al.	The journal of Hospital Infection	2021	Retrospective observational	ITALY	2006-2019	156	2019	Multicentre	This study aimed to identify and predict patients at higher risk of HAIs according to their characteristics on ICU admission by variables that are collected easily and routinely (as patients' demographics, origin and type of admission, medical history and disease severity) and to compare the performance of this new model with other early warning scores, including the Acute Physiologic Assessment and Chronic Health Evaluation II score and the Sequential Organ Failure Assessment score	General Hospital	ICU	Machine Learning	The Support Vector Machines (SVM) algorithm was applied to classify patients according to sex, patient origin, non-surgical treatment for acute coronary disease, surgical intervention, SAPS II at admission, presence of invasive devices, trauma, impaired immunity, antibiotic therapy in 48 hours before ICU admission	Surveillance	Healthcare-associated infections UTI		Identify patients who need more attention because of their high risk of HAIs so to reduce mortality, hospital stays and assistance costs				No	7827	4743	69 (median)				*PROS:* The authors' approach must not be could be integrated and/or modified according to specific needs.	Adapted Quality Assessment Tool for Retrospective and prospective observational studies	High Quality
35318153	Using Preoperative and Intraoperative Factors to Predict the Risk of Surgical Site Infections After Lumbar Spinal Surgery: A Machine Learning-Based Study	Liu et al.	World Neurosurgery	2022	Retrospective observational	China	Dec-10 Dec-19	109	2019	Single	Develop a machine learning model and a web predictor for predicting SSI in patients after Lumbar Spinal Surgery (LSS).	University	Other	Machine Learning	Through different training set, the XGB model was identifies as the best prediction model. In the XGB model, the importance of features was sorted in descending order as follows: surgical duration, age, BMI, estimated blood loss, number of operative segments, previous spinal surgery history, hypertension, and DM. Based on the best predictive performance of the XGB model, a web predictor was developed to predict SSI after posterior LSS.	Surveillance	SSI		Reduce the incidence of SSI after LSS		The model lacks validation from external cohorts		No	288		55.4 (mean)					Adapted Quality Assessment Tool for Retrospective and prospective observational studies	Low Quality

Figure S1: Digital technologies for country study

	Digital technology classification						
NUMBER OF ARTICLES INCLUDED	Digital health/e-health/m-health	Electronic health records (EHRs)	Health informatics	Machine Learning	Natural Language processing	Smartphone and tablet computing devices	Grand Total
Canada				2			2
China				3			3
France			1	1			2
Germany			2	1			3
Italy			1	1			2
Multicountry				1			1
Netherlands	1						1
Norway			1				1
Pakistan				1			1
Rwanda				1			1
Spain				1			1
Sweden				2	2		4
Switzerland				1			1
Thailand				1			1
UK	1		1			3	5
US	1	1	1	8	3		14
Grand Total	3	1	7	24	5	3	43

Figure S2: Key function for country study

Number of articles included	Public health key function	
	Outbreak detection	Surveillance
Canada		2
China		3
France		2
Germany	1	2
Italy		2
Multicountry	1	
Netherlands		1
Norway	1	
Pakistan		1
Rwanda		1
Spain		1
Sweden	1	3
Switzerland	1	
Thailand		1
UK		5
US	1	13

Figure S3: Digital technologies for microorganism and AMR

	Studies included	Digital technology classifications					
		Digital health/e-health/m-health	Electronic health records (EHRs)	Health informatics	Machine Learning	Natural Language processing	Smartphone and tablet computing devices
Individually named or groups of infectious diseases	Antimicrobial resistant organisms			3	3		
	COVID-19			1	1		
	Pseudomonas				1		
	VRE				1		
	Unknown	3	1	3	18	5	3
